# Virtual Reality for Patients With Chronic Musculoskeletal Pain and Disability: An Umbrella Review of Systematic Reviews

**DOI:** 10.1002/hsr2.71163

**Published:** 2025-08-12

**Authors:** Fahad Salman Alotibi, Walid Mohammed, Paul Hendrick, Fiona Moffatt

**Affiliations:** ^1^ Department of Physiotherapy, College of Applied Medical Sciences Taif University Taif City Makkah Province Kingdom of Saudi Arabia; ^2^ Department of Physiotherapy and Rehabilitation Sciences, School of Medical and Health Sciences University of Nottingham Nottingham UK

**Keywords:** chronic, exergame, immersive, low back pain, musculoskeletal, neck, pain, virtual reality

## Abstract

**Background and Aim:**

Virtual reality (VR) has been proposed for the management of chronic musculoskeletal pain (MSKP). This umbrella review aimed to systematically search, critically appraise, summarize, and synthesize the current systematic reviews (SRs) on delivering VR interventions to rehabilitate patients with chronic primary MSKP and disability.

**Method:**

Data were obtained from five databases. Only SRs were included. This umbrella review utilized the AMSTAR‐2 to assess the methodological quality of the included SRs and the GRADE to assess the certainty in the body of evidence.

**Results:**

Seven SRs were included. The overall confidence in the SRs ranged from low to critically low, whereas the certainty in the body of evidence ranged from moderate to very low. Whilst the majority of the SRs suggested that VR, standalone or adjunctive to other interventions, had a significant short‐term positive effect on patient‐reported outcomes for pain in patients with chronic primary MSKP, results on patient‐reported outcomes for disability and kinesiophobia were inconsistent. Adverse events included motion sickness, nausea, and vertigo.

**Conclusions:**

Although the current evidence indicates that VR may hold promise in patients with chronic primary MSKP, the included studies suffered from critical weaknesses that precluded this review from drawing a conclusive conclusion. It remains uncertain which VR interventions, including dosage, mode of delivery, supervision, frequency, duration, level of immersion, VR platform, displayed content, and mechanism of action, are more effective than the others. Future SRs should sub‐group VR based on the treatment types. Further rigorously designed studies focusing on immersive VR, standalone or adjunctive to other interventions, with long‐term follow‐up, are warranted. It is worth repeating the call for an agreed consensus on a clear definition and classification of VR within the healthcare context.

## 1 Introduction

Musculoskeletal (MSK) conditions are the most significant contributor to years lived with disability [1]. Chronic MSK pain (MSKP) is defined as pain that originates from the bones, joints, and muscles, which lasts for a minimum of 12 weeks [2]. Chronic primary MSKP is frequently characterized by functional disability, which is defined according to the International Association for the Study of Pain (IASP) [2] “interference in daily life activities and reduced participation in social roles” (p.87). In addition, the World Health Organization [3] has defined rehabilitation as “a set of interventions designed to optimize functioning and reduce disability in individuals with health conditions in interaction with their environment” (p.9). Chronic primary MSKP, as classified by the IASP, includes cervical, thoracic, low back, and limb pain [4]. The incidence and prevalence of MSK disorders are significant worldwide [5, 6].

The traditional management options for MSKP have been challenged by high costs [7] and patient adherence to treatment [8, 9], which may play an important role in the effectiveness of treatment [10, 11]. Therefore, there is a growing interest in evaluating promising novel, technology‐based approaches, such as virtual reality (VR), to address MSKP, disability, and kinesiophobia [12, 13, 14]. In addition, it was suggested that recent portable VR devices are a one‐time cost and may be continually used in clinics or homes [12, 15]. These advantages of VR may play an important role in addressing high cost and adherence while also enhancing the user experience within a gamified VR environment.

VR solutions have been proposed for pain management and have shown promising results in terms of pain and functional outcomes in various chronic pain conditions [16]. Furthermore, the mechanism of VR in patients with MSKP may include distraction [17], alleviating fear of movement [18], and altering body perception [19, 20]. In addition, the effectiveness of VR on patient‐reported outcomes in patients with MSKP has been extensively reviewed [12, 15, 21, 22, 23, 24, 25, 26, 27, 28, 29, 30, 31, 32]. Thus, an umbrella review intends to integrate and summarize evidence from several systematic reviews (SRs) of interventions for health conditions into a single, accessible, and usable synthesis [33, 34]. Therefore, the present umbrella review aimed to systematically search, critically appraise, summarize, and synthesize SRs on delivering VR interventions to rehabilitate patients with chronic primary MSKP and disability.

## 2 Methods

This umbrella review followed the Preferred Reporting Items for Systematic Reviews and Meta‐Analyses (PRISMA) guidelines [35]. Sources were systematically gathered using a predefined PICOS format, including:

- Population: adults with chronic primary MSKP;
- Intervention: VR;
- Comparison: any;
- Outcome: pain and disability as a primary and kinesiophobia and adverse events as a secondary), and;
- Study design (i.e., SR with/without meta‐analysis).

Duplicates were removed using EndNote version X9. This umbrella review was registered in the International Prospective Register of SRs (PROSPERO: CRD42022329517). The umbrella review has different terminologies, such as an overview of SRs, a review of reviews, and an SR of SRs [33, 34].

### 2.1 Shifting From Published Protocol

Studies may need to be updated frequently, and reviewers may shift from their protocols. In this umbrella review, the protocol detailed that one independent author (F.A.) would proceed with the umbrella review process in collaboration with the supervisory team (P.H. and F.M.). However, after submitting the protocol, an independent reviewer (W.M.) was identified to assist in completing this umbrella review. In addition, during the initial search in 2022, the anticipation was to identify only a few SRs focusing on chronic primary MSKP. However, upon piloting the search for the update in 2024, several SRs were identified. Therefore, including non‐chronic primary MSKP SRs would add unnecessary heterogeneity within the review's population. Furthermore, this umbrella review is part of a PhD thesis focusing on chronic primary MSKP, particularly CLBP. Therefore, a pragmatic decision was made to adhere to the IASP classification for chronic primary MSKP [4]. As a result, non‐chronic primary MSKP SRs were excluded.

### 2.2 Search Strategy

A comprehensive search strategy was developed after consultations with two librarians, “Tables in Supporting Information S1”. Initially, eligible SRs were limited to those published within the last 12 years (i.e., 10 years before being updated in 2024) owing to the advancements in VR technology [36, 37]. The search was performed on 9–10 April 2022, and updated on July 3, 2024. Following the PRISMA guidelines, the following databases were searched to identify eligible studies: Scopus, Web of Science, Cumulative Index to Nursing and Allied Health Literature, Cochrane Library (Cochrane Database of Systematic Reviews), and Ovid (i.e., MEDLINE, Allied and Complementary Medicine Database, and Embase). The reference lists of all the identified SRs were also assessed for eligibility.

### 2.3 Inclusion Criteria

The inclusion criteria (Table 1) were as follows: SRs assessing adults aged > 18 years with chronic primary MSKP and interactive VR. The IASP's classification for chronic primary MSKP was utilized for inclusion and exclusion criteria [4]. SR reports include a research question, searched sources, search strategy, inclusion and exclusion criteria, screening methods, critical appraisal of the included studies, assessment report, and information about data analysis and synthesis [38]. In addition, SRs utilize a systematic, transparent, and reproducible method to collect data from primary studies and synthesize findings with or without meta‐analysis [39]. VR is a computer‐generated technology that allows the user to have a sense of presence in a virtual environment and to interact with it [40]. VR can be classified as non‐immersive or immersive according to the level of immersion [16, 41, 42]. Immersive VR can be displayed on a head‐mounted display (HMD) that covers all the user's points of view [40]. Non‐immersive VR can be displayed on a screen or eyeglasses that do not cover all the user's points of view [41].

**Table 1:** Inclusion and exclusion criteria.

Inclusion criteria	Exclusion criteria
Adults aged > 18 years.	Non‐adult aged < 18 years.
Chronic primary musculoskeletal pain.	Non‐chronic primary musculoskeletal pain or postoperative pain.
Systematic reviews.	Primary studies and other types of review articles.
Interactive virtual reality.	Noninteractive VR, including mHealth, eHealth, self‐management applications, internet‐based, and videoconferencing technologies.
Access to full‐text with adequate information.	Unable to access full‐text or lack of enough information of interest.
Published since 2012.	Published before 2012.
English language.	Non‐English.

The primary outcomes were pain and disability; the secondary outcomes were kinesiophobia and adverse events. Kinesiophobia is widely known as a fear of movement [43]. Adverse events are defined as harmful or unpleasant reactions resulting from an intervention [44]. The settings included hospitals, clinics, rehabilitation centers, and university laboratories. The language was set to English because of a lack of funds for interpreters.

### 2.4 Exclusion Criteria

The exclusion criteria (Table 1) were as follows: primary studies, other types of review articles, letters, practice guidelines, and SRs assessing non‐chronic primary MSKP (i.e., chronic secondary MSKP [2]) or postoperative pain were excluded. In addition, noninteractive VR technologies, such as mHealth (e.g., self‐management applications) and eHealth (e.g., Internet‐based or videoconferencing technologies), were excluded.

### 2.5 Study Selection

In the first phase, two independent reviewers (F.A. and W.M.) screened the eligible studies based on their titles and abstracts. In the second phase, two reviewers (F.A. and W.M.), under the supervisory team (P.H. and F.M.), independently performed full‐text screening to decide whether to include or exclude the selected articles. Any disagreements were resolved by a discussion among the reviewers.

### 2.6 Data Extraction

Two independent authors (F.A. and W.M.) extracted the data from each SR included in this study. Any differences in opinions were resolved by a discussion that reached an agreement. If further information was required, the corresponding author of the specific study was contacted. An amended JBI data extraction form for umbrella reviews was used to extract the relevant data (described in “Supporting Information S2”). The timeframe for outcome measurement includes short‐term follow‐up (close to 4 weeks), intermediate‐term follow‐up (close to 24 weeks), and long‐term follow‐up (close to 12 months) [45].

### 2.7 Quality Assessment

This umbrella review utilized A MeaSurement Tool to Assess SRs—2 (AMSTAR‐2) to assess the methodological quality of the included SRs [46] and the Grading of Recommendations, Assessment, Development, and Evaluations (GRADE) to assess the certainty in the body of evidence [47] (described in “Supporting Information S2”). Two reviewers (F.A. and W.M.) critically appraised the selected SRs. A discussion among the reviewers resolved any disagreements. The results of the risk of bias and the quality assessment from the included SRs were summarized and reported in “Supporting Information S3: Tables 1 and 2”.

**Table 2:** Characteristics of the included systematic reviews.

Authors	Conditions	Number of studies	Total number of participants in the primary studies included in the reviews	Settings
Brea‐Gómez et al. (2021)	Chronic low back pain (CLBP)	14 Randomized controlled trials (RCTs)	765 (62.08% male); mean age of participants: 40.04	Not reported (NR)
Grassini (2022)	Chronic neck pain (CNP) and CLBP)	9 RCTs	524 participants (sex and average age: NR)	NR
Hao et al. (2024)	CNP	6 RCTs	243 participants (sex and average age: NR)	Five RCTs in clinical settings and one at patients' homes.
Kumar et al. (2024)	CLBP	7 RCTs	507 participants (sex: NR); average age: 48.4 years	NR
Li et al. (2024)	CLBP	20 RCTs	1059 participants (sex: 44.9% male, 2.4% NR); average age: NR	NR
Ye et al. (2023)	CNP	5 (Four RCTs and one feasibility study)	192 (sex: NR in numbers); average age: NR	NR
Zhang et al. (2024)	CNP and CLBP	16 RCTs	800 (sex: total NR); average age: NR	NR

### 2.8 Overlapping Studies

Overlapping studies are primary studies cited in multiple SRs, resulting in double‐counting of data (i.e., duplicates) [48]. Some challenges can arise from double‐counting outcome data, which may provide misleading information, such as excessive influence and statistical weight or overestimating the intervention effect [49]. It might be common in umbrella reviews with several linked SRs that have similar research questions, where they examine the same intervention for the same disease and include several primary studies [49]. Therefore, the formula provided by Pieper [50] was used to calculate the corrected covered area (CCA) to account for overlapping studies. The CCA interpretations were as follows: 0–5, slight; 6–10, moderate; 11–15, high; and > 15, very high.

### 2.9 Data Synthesis

The planned protocol described data analysis as narrative syntheses used to summarise and synthesise outcomes. For an umbrella review, no data meta‐analysis was performed. The data are reported according to their presentation in SRs and meta‐analyses. The characteristics of the SRs are presented in the provided tables (described in “Supporting Information S2”). The rating assessment, including the overall confidence and certainty in the body of evidence in the results of the SRs, is reported in the provided tables.

## 3 Results

**Figure 1:**
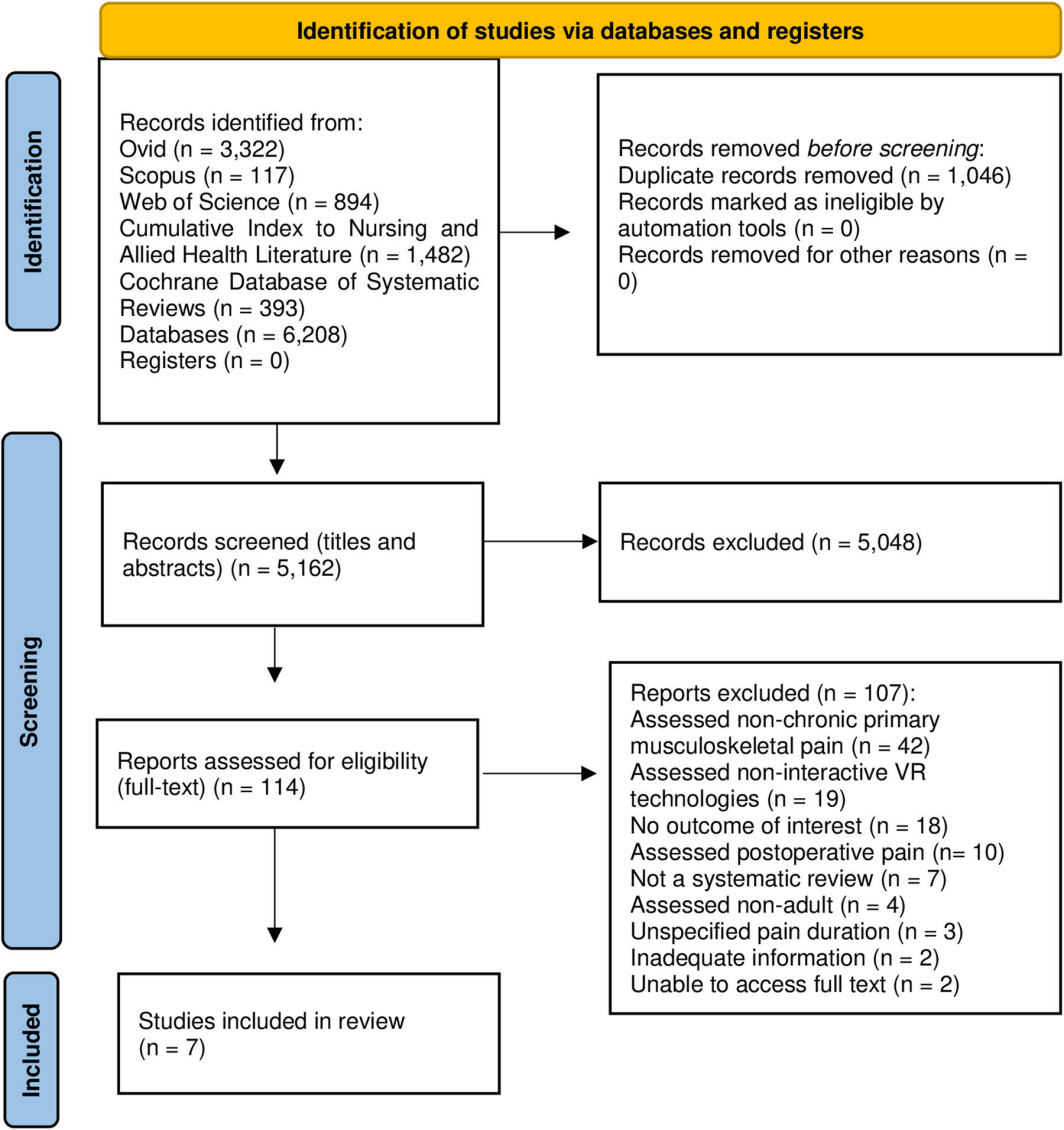
Preferred reporting items for systematic reviews and meta‐analyses flow diagram.

The search generated 6208 records from databases (Figure 1). After duplicates (*n* = 1046) were removed, the total number of records was reduced to 5162. Screening the titles and abstracts, 5048 records were removed, and 114 reports were screened for full text. The list of excluded studies with reasons (*n* = 107) is reported in “Supporting Information S4”. Therefore, seven SRs met the inclusion criteria in this umbrella review [21, 22, 51, 52, 53, 54, 55].

### 3.1 Study Characteristics

The seven SRs encompassed 76 randomized controlled trials (RCTs) and one feasibility study, out of which 36 were unique primary studies (Table 2). Two SRs focused on chronic spinal pain (CSP), including chronic neck pain (CNP) and CLBP [22, 55], two only assessed CNP [51, 54], and three only assessed CLBP [21, 52, 53].

### 3.2 Quality Assessment

The included SRs were assessed using AMSTAR‐2 (Table 3). The overall confidence in the results of the included SRs ranged from low to critically low. The GRADE assessment for each outcome ranged from moderate to very low and is presented in “Supporting Information S5: Table 1”.

**Table 3:** AMSTAR‐2 rating.

Authors	AMSTAR‐2 items																Overall confidence
	1	2	3	4	5	6	7	8	9	10	11	12	13	14	15	16	
Brea‐Gómez et al. (2021)	Y	PY	Y	PY	Y	Y	Y	PY	Y	N	Y	N	N	Y	N	Y	Critically low
Grassini (2022)	Y	N	Y	N	N	N	N	N	Y	N	Y	N	N	Y	Y	Y	Critically low
Hao et al. (2024)	Y	PY	N	N	Y	Y	N	PY	Y	N	N	Y	N	N	N	Y	Critically low
Kumar et al. (2024)	Y	N	N	N	Y	N	N	PY	Y	N	N	N	N	N	N	Y	Critically low
Li et al. (2024)	Y	Y	Y	N	N	Y	N	PY	Y	N	Y	N	N	Y	N	Y	Critically low
Ye et al. (2023)	Y	PY	N	N	Y	Y	N	PY	Y	N	Y	Y	Y	Y	N	Y	Critically low
Zhang et al. (2024)	Y	PY	N	Y	Y	Y	N	PY	Y	N	Y	Y	Y	Y	Y	Y	Low

Abbreviations: N, no; PY, partially yes; Y, yes.

### 3.3 Participants

In all eligible studies [21, 22, 51, 52, 53, 54, 55], the participants were adults aged > 18 years (Table 2). The total number of participants was 4090 across all the SRs. Two SRs reported mean ages of 40.04 years and 48.4 years, respectively [21, 52]. Two SRs reported sex, including a predominant female participant in one (44.9% male) [53] but not in the other (62.08% male) [21]. However, two SRs reported sex in percentages for each primary study [54, 55], which was not clear regarding the actual number of participants. Sex was not reported in three SRs [22, 51, 52].

### 3.4 Virtual Reality Groups

Each unique primary study (*n *= 36) was presented once to avoid repetition of VR interventions across SRs due to overlapping “Table in Supporting Information S6”. VR was studied at different levels of immersion, including immersive (*n* = 11) and non‐immersive (*n* = 25) (described in “Supporting Information S2”).

### 3.5 Control Groups

Similar to VR groups, each primary study was presented once to avoid repetition “Table in Supporting Information S6”. Control groups received various types of interventions (described in “Supporting Information S2”).

### 3.6 Duration of Virtual Reality Rehabilitation

The duration of VR rehabilitation for each primary study is reported in the “Table in Supporting Information S6”. The rehabilitation duration varied across the primary studies, and the duration of the sessions was heterogeneous. The duration of the VR sessions ranged from 2‐ to 60‐min, and the frequency of the sessions per week ranged from one session to seven sessions per week, with the rehabilitation duration ranging from 1 to 8 weeks. The number of sessions ranged from a single session to 56.

### 3.7 Countries and Settings of Included Primary Studies

All countries of the primary studies across the included SRs are presented in the “Table in Supporting Information S6”. Only one of the included SRs provided information regarding the study settings [51], which included clinical settings and patients' homes (Table 2). However, the countries of the included studies were reported in four SRs “Table in Supporting Information S6 and described in Supporting Information S2” [21, 51, 53, 55]. Therefore, primary studies had been searched to investigate the countries of the studies that were not reported by Grassini [22], Kumar et al. [52], and Ye et al. [54].

### 3.8 Outcomes

In this umbrella review, pain and disability were the primary outcomes, while kinesiophobia and adverse events were the secondary outcomes. Meta‐analysis was pooled by all the included SRs. The summarized results of all meta‐analyses with certainty in the body of evidence using GRADE are in the Supporting Information S5: Table 1, whereas the summarized main findings of the meta‐analyses are presented in Table 4. The timeframe was not precisely specified by all SRs for each individual meta‐analysis [21, 22, 52, 54, 55]. However, short‐term and intermediate‐term follow‐ups reported in some meta‐analyses [21, 51, 53]. No SRs reported long‐term follow‐up. Primary and secondary outcome measures were reported in Supporting Information S2.

**Table 4:** Main findings of included systematic reviews.

Authors	Condition “comparisons”	Outcome “measure, score”	Trials (participants)	Effect estimates 95% CI, *I* ^2^, *p* value	Certainty in the body of evidence
Brea‐Gómez et al. (2021)	Chronic low back pain (CLBP): “Virtual reality (VR) vs. control”	Pain: “Visual Analogue Scale (VAS), 0–10”	11 (569)	(Standardized mean difference [SMD] = −1.92, 95% confidence interval [CI] = −2.73 to −1.11, *I* ^2^ = 74%, *p* < 0.00001), in favor of the VR group (VRG).	Low
		Disability: “Oswestry Disability Index (ODI), not reported (NR)”	3 (147)	(Mean difference [MD] = −10.46, 95% CI = −30.02 to 9.09, *I* ^2^ = 99%, *p* = 0.29), no statistically significant difference.	Very low
		Kinesiophobia: “Tampa Scale of Kinesiophobia (TSK)−17, NR”	3 (192)	(MD = −8.96, 95% CI = −17.52 to –0.40, *I* ^2^ = 99%, *p* = 0.04), in favor of VRG.	Very low
Grassini (2022)	Chronic neck pain (CNP): “VR vs. control”	Pain: “VAS, 0–100”	2 (146)	(MD = −8.80, 95% CI = −12.49 to −3.69, *I* ^2^ = 0%, *p* < 0.05), in favor of VRG.	Low
		Disability “Neck Disability Index (NDI), 0–50”	3 (136)	(MD = −2.87, 95% CI = −4.36 to −1.39, *I* ^2^ = 33%, *p* = 0.0002), in favor of VRG.	Low
		Kinesiophobia: “TSK, 0–68”	2 (94)	(MD = −0.28, 95% CI = −3.46 to −2.90, *I* ^2^ = 0%, *p* = 0.85), no significant difference.	Low
	CLBP: “VR vs. control”	Pain: “VAS, 0–100”	4 (158)	(MD = −10.15, 95% CI = −23.42 to 3.12, *I* ^2^ = 95%, *p* = 0.13), no significant difference.	Very low
		Disability: “ODI, 0–100”	2 (66)	(MD = −0.67, 95% CI = −7.81 to −6.46, *I* ^2^ = 73%, *p* = 0.85), no significant difference.	Very low
		Kinesiophobia: “TSK, 0–68”	3 (136)	(MD = −9.77, 95% CI = −21.43 to −1.88, *I* ^2^ = 98%, *p* = 0.10), no significant difference.	Very low
	Chronic spinal pain (CSP): “VR vs. control”	Pain: “VAS, 0–100”	7 (147)	(MD = −9.10, 95% CI = −17.64 to −0.57, *I* ^2^ = 92%, *p* = 0.04), in favor of VRG.	Very low
		Kinesiophobia: “TSK, 0–68”	5 (130)	(MD = −6.00, 95% CI = −14.57 to 2.57, *I* ^2^ = 97%, *p* = 0.17), no significant difference.	Very low
Hao et al. (2024)	CNP: “VR vs. control; at short term”	Pain: “VAS, 0–10”	6 (275)	(MD = −0.94, 95% CI = −1.31 to −0.58, *I* ^2^ = 0%, *p* < 0.01), in favor of VRG.	Low
		Disability “NDI, 0–50”	5 (241)	(MD = −2.16, 95% CI = −3.50 to −0.82, *I* ^2^ = 15.4%, *p* < 0.01), in favor of VRG.	Low
		Kinesiophobia “TSK, NR”	3 (166)	(MD = −1.04, 95% CI = −3.54 to 1.45, *I* ^2^ = 0%, *p* = 0.41), no significant difference.	Low
Kumar et al. (2024)	CLBP: “VR vs. control”	Pain: “NR, NR”	7 (507)	(MD = 1.99, 95% CI = 0.60–3.38, *I* ^2^ = 95%, *p* < 0.005), in favor of VRG.	Low
Li et al. (2024)	CLBP: “VR vs. control; at short term”	Pain: “VAS, 11‐point Numerical Pain Rating scale, Defense and Veterans Pain Rating Scale, 0–10”	19 (879)	(MD = −1.43, 95% CI = −1.86 to −1.00, *I* ^2^ = 95%, *p *< 0.001), in favor of VRG.	Low
		Disability “ODI, NR”	8 (377)	(MD = −11.50, 95% CI = −20.00 to −3.01, *I* ^2^ = 95%, *p* = 0.008), in favor of VRG.	Very low
		Kinesiophobia “TSK, NR”	6 (229)	(MD = −5.46, 95% CI = −9.40 to −1.52, *I* ^2^ = 90%, *p* = 0.007), in favor of VRG.	Very low
Ye et al. (2023)	CNP: “VR vs. control”	Pain: “VAS, NR”	4 (155)	(MD = 0.58, 95% CI = 0.25–0.91, *I* ^2^ = 2%, *p* = 0.0005), in favor of VRG.	Moderate
		Disability “NDI, NR”	3 (121)	(MD = 0.54, 95% CI = −0.15 to 1.24, *I* ^2^ = 72%, *p* = 0.13), no significant difference.	Low
Zhang et al. (2024)	CNP: “VR vs. control”	Pain: “VAS, 0–10”	4 (180)	(Weighted MD [WMD] = −1.22, 95% CI = −1.80 to −0.63, *I* ^2^ = 0%, *p *< 0.00001), in favor of VRG.	Low
	CLBP: “VR vs. control”	Pain: “VAS, 0–10”	11 (596)	(WMD = −1.74, 95% CI = −2.28 to −1.20, *I* ^2^ = 92%, *p *< 0.00001), in favor of VRG.	Low
	CSP: “VR vs. control group”	Pain: “VAS, 0–10”	15 (776)	(WMD = −1.63, 95% CI = −2.11 to −1.20, *I* ^2^ = 90%, *p *< 0.001), in favor of VRG.	Low
		Disability “NDI, NR”	3 (139)	(WMD = −2.66, 95% CI = −5.47 to −0.15, *I* ^2^ = 48%, *p* = 0.06), no significant difference.	Low
		Kinesiophobia “TSK‐17, NR”	2 (96)	(WMD = −9.66, 95% CI = −22.01 to 2.68, *I* ^2^ = 97%, *p* = 0.13), no significant difference.	Low
	CSP: “Immersive VR vs. control group”	Pain: “VAS, 0–10”	7 (406)	(WMD = −1.50, 95% CI = −2.45 to −0.55, *I* ^2^ = 80%, *p *< 0.001), in favor of VRG.	Low
	CSP: “Non‐immersive VR vs. control group”	Pain: “VAS, 0–10”	8 (370)	(WMD = −1.79, 95% CI = −2.31 to −1.27, *I* ^2^ = 91%, *p *< 0.001), in favor of VRG.	Low

#### 3.8.1 Primary Outcomes

##### 3.8.1.1 Pain

In patients with CNP, based on moderate to low certainty in the body of evidence, four SRs demonstrated a statistically significant difference in favor of VR compared with control in reducing pain outcomes: (mean difference [MD] = −8.80, 95% confidence interval [CI] = −12.49 to −3.69, *I* ^2^ = 0%, *p* < 0.05) [22], (MD = −0.94, 95% CI = −1.31 to −0.58, *I* ^2^ = 0%, *p* < 0.01) [51], (MD = 0.58, 95% CI = 0.25–0.91, *I* ^2^ = 2%, *p* = 0.0005) [54], and (weighted MD [WMD] = −1.22, 95% CI = −1.80 to −0.63, *I* ^2^ = 0%, *p *< 0.00001) [55].

In patients with CLBP, based on low certainty in the body of evidence, four SRs demonstrated a statistically significant difference in favor of VR compared with control or no VR intervention groups in reducing pain outcomes (standardized MD [SMD] = −1.92, 95% CI = −2.73 to −1.11, *I* ^2^ = 74%, *p* < 0.00001) [21], (MD = 1.99, 95% CI = 0.60 to 3.38, *I* ^2^ = 95%, *p* < 0.005) [52], (MD = −1.43, 95% CI = −1.86 to −1.00, *I* ^2^ = 95%, *p *< 0.001) [53], and (WMD = −1.74, 95% CI = −2.28 to −1.20, *I* ^2^ = 92%, *p *< 0.00001) [55]. In contrast, based on very low certainty in the body of evidence, one SR demonstrated no statistically significant difference between VR and control groups in reducing pain outcomes in patients with CLBP (MD = −10.15, 95% CI = −23.42 to 3.12, *I* ^2^ = 95%, *p* = 0.13) [22].

In patients with CSP, based on low to very low certainty in the body of evidence, two SRs demonstrated a statistically significant difference in favor of VR compared with control in reducing pain outcomes (MD = −9.10, 95% CI = −17.64 to −0.57, *I* ^2^ = 92%, *p* = 0.04) [22] and (WMD = −1.63, 95% CI = −2.11 to −1.20, *I* ^2^ = 90%, *p *< 0.001) [55]. In addition, based on low certainty in the body of evidence, one SR demonstrated a statistically significant difference in favor of immersive VR compared with control in reducing pain outcomes in patients with CSP (WMD = −1.50, 95% CI = −2.45 to −0.55, *I* ^2^ = 80%, *p *< 0.001) [55]. Furthermore, based on very low certainty in the body of evidence, one SR demonstrated a statistically significant difference in favor of non‐immersive VR compared with control in reducing pain outcomes in patients with CSP (WMD = −1.79, 95% CI = −2.31 to −1.27, *I* ^2^ = 91%, *p *< 0.001) [55].

##### 3.8.1.2 Disability

In patients with CNP, based on low certainty in the body of evidence, two SRs demonstrated a statistically significant difference in favor of VR compared with control in reducing disability outcomes (MD = −2.87, 95% CI = −4.36 to −1.39, *I* ^2^ = 33%, *p* = 0.0002) [22] and (MD = −2.16, 95% CI = −3.50 to −0.82, *I* ^2^ = 15.4%, *p* < 0.01) [51]. In contrast, based on low certainty in the body of evidence, one SR demonstrated no statistically significant difference between VR and control groups in reducing disability outcomes (MD = 0.54, 95% CI = −0.15 to 1.24, *I* ^2^ = 72%, *p* = 0.13) [54].

In patients with CLBP, based on very low certainty in the body of evidence, one SR demonstrated a statistically significant difference in favor of VR compared with control in reducing disability outcomes (MD = −11.50, 95% CI = −20.00 to −3.01, *I* ^2^ = 95%, *p* = 0.008) [53]. In contrast, based on very low certainty in the body of evidence, two SRs demonstrated no statistically significant difference between VR and control or no VR intervention groups in reducing disability outcomes in patients with CLBP (MD = −10.46, 95% CI = −30.02 to 9.09, *I* ^2^ = 99%, *p* = 0.29) [21] and (MD = −0.67, 95% CI = −7.81 to −6.46, *I* ^2^ = 73%, *p* = 0.85) [22].

In patients with CSP, based on low certainty in the body of evidence, one SR demonstrated no statistically significant difference between VR and control groups in reducing disability outcomes (WMD = −2.66, 95% CI = −5.47 to −0.15, *I* ^2^ = 48%, *p* = 0.06) [55].

#### 3.8.2 Secondary Outcomes

##### 3.8.2.1 Kinesiophobia

In patients with CNP, based on low certainty in the body of evidence, two SRs demonstrated no statistically significant difference between VR and control groups in reducing kinesiophobia outcomes (MD = −0.28, 95% CI = −3.46 to −2.90, *I* ^2^ = 0%, *p* = 0.85) [22] and (MD = −1.04, 95% CI = −3.54 to 1.45, *I* ^2^ = 0%, *p* = 0.41) [51].

In patients with CLBP, based on very low certainty in the body of evidence, two SRs demonstrated a statistically significant difference in favor of VR compared with control or no VR intervention in reducing kinesiophobia outcomes (MD = −8.96, 95% CI = −17.52 to –0.40, *I* ^2^ = 99%, *p* = 0.04) [21] and (MD = −5.46, 95% CI = −9.40 to −1.52, *I* ^2^ = 90%, *p* = 0.007) [53]. In contrast, based on very low certainty in the body of evidence, one SR demonstrated no statistically significant difference between VR and control groups in reducing kinesiophobia outcomes in patients with CLBP (MD = −9.77, 95% CI = −21.43 to −1.88, *I* ^2^ = 98%, *p* = 0.10) [22].

In patients with CSP, based on very low certainty in the body of evidence, two SRs demonstrated no statistically significant difference between VR and control groups in reducing kinesiophobia outcomes (MD = −6.00, 95% CI = −14.57 to 2.57, *I* ^2^ = 97%, *p* = 0.17) [22] and (WMD = −9.66, 95% CI = −22.01 to 2.68, *I* ^2^ = 97%, *p* = 0.13) [55].

##### 3.8.2.2 Adverse Events

Three SRs considered adverse events, two of which reported nausea and motion sickness [21, 51, 55] and one reported vertigo [21]. Four SRs did not consider adverse events [22, 52, 53, 54].

### 3.9 Overlapping Studies

The overlapping primary studies across the included SRs are presented in “Table in Supporting Information S7”. The formula provided by Pieper [50] was used ([77 − 36]/([36 × 7] − 36) = 41/216 = 0.1898). Therefore, the CCA is 18.98%, which indicates a very high overlap. The overlapping percentage indicates that it more than likely reflects an unnecessary duplication between the included SRs.

## 4 Discussion

This is the first umbrella review in the field of MSK that comprehensively searched, critically appraised, summarized, and synthesized SRs studying VR interventions to rehabilitate patients with chronic primary MSKP and disability. The protocol has been registered prospectively, and the deviation from the registered protocol was discussed. Furthermore, no SRs offered a high certainty in the body of evidence or long‐term follow‐up to underpin VR's effectiveness on patient‐reported outcomes for pain, disability, and kinesiophobia in patients with chronic primary MSKP. The range of VR intervention platforms, dosage, delivery, settings, and the countries where the VR was delivered have been described. Furthermore, this umbrella review included SRs that evaluated VR standalone and as an adjunct to other treatment approaches (e.g., physiotherapy care). This emphasizes the role of a set of interventions that align with the WHO definition of rehabilitation [3].

### 4.1 Summary of Findings

Overall, based on low to moderate certainty in the body of evidence, 12 meta‐analyses' results suggested that VR, compared to control, had a significant short‐term positive effect on pain outcome measures in patients with chronic primary MSKP. These findings are consistent with previous SRs of VR on patient‐reported outcomes for pain in patients with MSKP [15, 23, 28, 29, 31, 32, 42].

However, based on very low certainty in the body of evidence, one meta‐analysis suggested that the effectiveness of VR on patient‐reported outcomes for pain in patients with CLBP was inconsistent. This rather contradictory result is consistent with an earlier SR [24]. Inconsistency may be attributed to the heterogeneity of the VR and control group interventions and the variations in the certainty of the body of evidence among included studies.

In this umbrella review, based on very low to low certainty in the body of evidence, the meta‐analyses' results for disability and kinesiophobia outcome measures were contradictory. Recent SRs' results were also not consistent in terms of the effectiveness of VR on disability outcomes in patients with CLBP [29, 53] and kinesiophobia outcomes in patients with CNP [32, 42]. Despite the heterogeneity of the VR intervention duration and platforms included in all SRs, a possible explanation for this discrepancy might be that there were differences in the sample size and the number of studies included. Adverse events were reported as motion sickness, nausea, and vertigo. Overall, there was inconsistency in reporting adverse events among SRs, as some considered reporting but did not report all events. For example, two primary studies by Bahat et al. [56] and Monteiro‐Junior et al. [57] reported motion sickness and vertigo, respectively, which were reported in one SR [51] but not in the other SR that considered reporting adverse events [55].

### 4.2 Limitations of Included Systematic Reviews

The overall confidence in the included SRs ranged from low to critically low based on AMSTAR‐2 [46] assessment. There were some limitations in describing the intervention, which may be attributed to reporting limitations in primary studies. Furthermore, it is worth re‐emphasizing that only one of the included SRs reported a list of the excluded primary studies [21]. Thus, future SRs are encouraged to provide a comprehensive list of excluded studies and a clear justification for each exclusion to enhance transparency.

Moreover, one of the included SRs utilized only one independent reviewer [22], it is recommended that reviewers perform a double data extraction to decrease the chance of errors [58]. In addition, despite the very high overlap between SRs, there were also variations in assessing the methodological quality of evidence. Although the risk of bias tools have been recognized as subjective [59, 60], this may have led to overrating the certainty in the body of evidence results. For example, all RCTs included in Ye et al. [54] had a low risk of bias in all items but were found to have at least one item with a high risk of bias when assessed by other included SRs [22, 51, 55]. As a result, Ye et al. [54] meta‐analysis was not downgraded for risk of bias, and was the only result with moderate certainty in the body of evidence. Furthermore, this limitation may extend to be a limitation of this umbrella review. Thus, future SRs are encouraged to employ a robust technique when assessing the risk of bias and also to consider this risk when interpreting the results.

The included SRs lacked a detailed description of the included VR intervention, highlighting the need for a standardized reporting framework for VR intervention among primary studies. Although one of the included SRs conducted a subgroup analysis based on the level of immersion [55], there was still variability within the VR protocols. In addition, SRs lacked a clear definition of VR and its categories. This may be attributed to the subjectivity and complexity of VR classification, highlighting the need to unify the definition and classification of VR within the healthcare context [61].

### 4.3 Limitations of This Umbrella Review

The limitations of this study are that some SRs might not have been identified since the language was set to English, and the search filter on databases was set to reviews. Moreover, considering the aim of this umbrella review, assessing the publication bias or risk of bias in primary studies is beyond the scope of this umbrella review, as this might significantly increase the amount of time and effort required to complete the umbrella review [62].

The search date was restricted to 12 years (i.e., 10 years before being updated in 2024) due to advancements in VR technologies. Although all evidence should be considered, this significant development in VR may render some evidence less relevant due to rapid technological advancement. For instance, the latest identified version of immersive VR from Meta was Quest [63], while the available on‐the‐market version is Quest 3S. Although this area of literature has developed rapidly in the past few years, technological advancements seem to be evolving even faster.

The VR interventions included in this umbrella review were not homogeneous. Although the VR hardware in this study was categorized according to the level of immersion (i.e. immersive, non‐immersive), the VR intervention platforms and protocols varied. Most of the included SRs mixed immersive and non‐immersive VR when pooling the evidence and, therefore, complicated the evidence synthesis of this umbrella review.

Therefore, combining various VR types and variability in methods and protocols may cast some doubt on which VR interventions, including dosage, mode of delivery, supervision, frequency, duration, level of immersion, VR platform, displayed content, and mechanism of action, are more effective than the others. This precluded the umbrella review from reaching a conclusive efficacy conclusion or providing meaningful cross‐study comparisons. However, this umbrella review underscores the gaps and variability in the methodological quality of VR studies in the field of chronic primary MSKP. Therefore, the results should be interpreted as a map of evidence gaps rather than a conclusive evaluation of VR's efficacy.

Although the IASP classification for chronic primary MSKP [4] was utilized to enhance the patient population's homogeneity, some level of heterogeneity in terms of severity and underlying causes remained. For example, one SR that reviewed CNP comprised six primary studies: four nonspecific, one traumatic, and one whiplash [51]. Therefore, limitations of the included SRs may have extended to this umbrella review owing to the very high duplication among the included SRs.

Although the Pieper et al. [50] formula indicated a very high overlapping of primary studies among the included SRs, this may suggest that the included SRs collectively represented a comprehensive overview of the existing literature. In addition, the limitation of overlapping primary studies has been recognized as a challenge when conducting an umbrella review [64], potentially overestimating the effect of an intervention [49]. Moreover, statistically pooling the retrieved meta‐analyses from the included SRs can increase the risk of such overlap [65], which this review endeavored to mitigate.

Nonetheless, the very high overlapping among included studies in this umbrella review may misrepresent the number of existing VR studies within the field of chronic primary MSKP. Therefore, each primary study was reported only once to clarify the number of existing primary studies. This approach attempts to mitigate the overlapping issue and avoid overstating the extent to which each VR intervention has been investigated.

### 4.4 Recommendations for Practice, Future Systematic Reviews and Primary Studies

It was not possible to draw a recommendation based on low to very low certainty in the body of evidence. Caution should be considered when interpreting the results, as the certainty in the body of evidence and the overall confidence in the SRs were mostly low to critically low. In addition, SRs need to consider the timeframe for each individual result. Future SRs are recommended to follow guidelines and checklists (such as PRISMA [“Supporting Information S8”] and AMSTAR‐2) to improve reporting quality. Furthermore, it is recommended that future SRs subgroup VR. In addition, the scores for each outcome measure may need to be reported to assist in interpreting the results. However, the clinical significance was not interpreted in line with another umbrella review in the pain context [66]. Such an interpretation may produce misleading information [67] and not necessarily represent the patients' perspective [68].

Future primary studies should follow a framework when designing and delivering an intervention, such as the Medical Research Council (MRC) framework for complex interventions [69] and the Virtual Reality Clinical Outcomes Research Experts (VR‐CORE) framework [70]. Moreover, to improve the quality of intervention descriptions, utilizing the Template for Intervention Description and Replication (TIDieR) framework is recommended [71]. In addition, there is an urgent need for more robust methodologies for conducting future primary studies.

Future primary studies are required to investigate immersive VR's feasibility, acceptance, and adverse events in other sociocultural contexts [27, 53]. Furthermore, the majority of the primary studies that combined VR with physiotherapy were limited to non‐immersive VR in patients with CLBP (*n* = 7). This raises intriguing questions regarding whether the emerging immersive VR, when combined with physiotherapy, produces better positive outcomes in patients with CLBP.

In the context of CLBP, further gaps regarding real‐life settings, the use of objective outcome measures, patient position and diagnosis, long‐term effect, cost‐effectiveness, a standardized protocol for VR, and treatment fidelity were identified. The results of this review highlight the need for further robust studies to investigate the effectiveness of immersive VR on pain, disability, and kinesiophobia outcomes.

All the SRs mentioned various potential mechanisms of VR analgesia, including distraction, improving fear of movement, and neuroplasticity. Furthermore, scoping and narrative reviews in the literature attempted to explain potential mechanisms [36, 72, 73]. However, mechanistic research into VR is warranted in this field to elucidate its impact on chronic primary MSKP.

## 5 Conclusion

Although the results suggest that VR, either as an alternative form of treatment or in combination with other interventions, may provide a short‐term positive impact on patient‐reported outcomes for pain in patients with chronic primary MSKP, it remains uncertain which specific VR intervention shows the most promise, as the included SRs grouped various types of VR together. The evidence is currently insufficient to make strong recommendations regarding the use of VR. Future robust primary and secondary studies focusing on immersive VR, standalone or adjunctive to other interventions, for patients with chronic primary MSKP are warranted.

## Author Contributions

**Fahad Salman Alotibi:** conceptualization, methodology, data curation, investigation, validation, visualization, writing – original draft, writing – review and editing. **Walid Mohammed:** data curation, validation, investigation. **Paul Hendrick:** conceptualization, methodology, validation, writing – review and editing, supervision. **Fiona Moffatt:** conceptualization, methodology, validation, supervision, writing – review and editing.

## Conflicts of Interest

The authors declare no conflicts of interest.

## Transparency Statement

The lead author, Fahad Salman Alotibi, affirms that this manuscript is an honest, accurate, and transparent account of the study being reported; that no important aspects of the study have been omitted; and that any discrepancies from the study as planned (and, if relevant, registered) have been explained.

## Supporting information

S1 File. Search Terms.

S2 File. Supplemental Methods and Results.

S3 File. Risk of Bias and Quality Assessment as Reported by Systematic Reviews.

S4 File. Excluded Studies.

S5 File. GRADE Results.

S6 File. Virtual Reality and Control Group Interventions.

S7 File. Overlapping Studies.

S8 File. PRISMA Checklists.

## Data Availability

Data sharing not applicable to this article as no data sets were generated or analyzed during the current study.
