# Supplementary material for: Virtual Reality for Patients With Chronic Musculoskeletal Pain and Disability: An Umbrella Review of Systematic Reviews
Source: Health Sci Rep. 2025 Aug 12;8(8):e71163. doi: 10.1002/hsr2.71163 (PMC12343317; doi:10.1002/hsr2.71163)
Supplement: Supplementary file 1 — S1 File. Search Terms. [file HSR2-8-e71163-s007.docx]

Databases: Ovid, Amed, Embase, Scopus, Web of Science, CINAHL, and The Cochrane Library (Cochrane Database of Systematic Reviews [CDSR]

The effectiveness of Virtual Reality on Musculoskeletal Pain: Systematic review of systematic reviews

Search terms for the search conducted on **9-10 April 2022**

|  | PICO format | Main concept | Synonyms |
| --- | --- | --- | --- |
| 1# | Population | Musculoskeletal Pain | Cervical, neck, back, low back, midthoracic, thoracic, lumbar, sacrum, sacral, sacroiliac, pelvic, shoulder, shoulder joint, shoulder impingement syndrome, subacromial, hip, hip osteoarthritis, knee joint, knee osteoarthritis, knee, osteoarthritis, arthritis, ankle, ankle joint, muscle, muscle*, muscular, bone, bony, joint, joint*, spondylosis, spondylolisthesis, ankylosing, spine, spinal, nociceptive, |
| 2# | Intervention | Virtual Reality | Virtual Reality, computer simulation, VR, postural balance, video games, Wii, Nintendo, Virtual Realit*, Exergaming, Video Games, Head Mounted Display, Kinect, Xbox, Equine-Assisted Therapy, Hippotherapy Simulator, Equine Simulator, Horseback, game, Games, gaming, Gamification, Mobile Application, mobile applications, PlayStation, Simulation Training, Simulation technologies, Simulation technology, Patient Simulation, virtual simulation, Simulation, Simulator, VR-based, Virtual reality based |
| 3# | Outcome | Pain and disability | Pain, chronic Pain, disability, ache, discomfort, Injury, injuries, injur*, spasm, sprain, strain, tenderness, contracture. |
| 3# | Combined 1# OR 2# and 3# | | |

| Scopus | "Pain" OR "chronic Pain" OR "chronic" OR "acute" OR "disability" OR "ache" OR "discomfort" OR "Injury" OR "injuries" OR "injur*" OR "spasm" OR "sprain" OR "strain" OR "tenderness" OR "contracture" OR "Cervical" OR "neck" OR "back" OR "low back" OR "midthoracic" OR "thoracic" OR "lumbar" OR "sacrum" OR "sacral" OR "sacroiliac" OR "pelvic" OR "shoulder" OR "shoulder joint" OR "shoulder impingement syndrome" OR "subacromial" OR "hip" OR "hip osteoarthritis" OR "knee joint" OR "knee osteoarthritis" OR "knee" OR "osteoarthritis" OR "arthritis" OR "ankle" OR "ankle joint" OR "muscle" OR "muscle"" OR "muscular" OR "bone" OR "bony" OR "joint" OR "joint"" OR "spondylosis" OR "spondylolisthesis" OR "ankylosing" OR "spine" OR "spinal" OR "nociceptive" OR "low back pain" AND "Virtual Reality" OR "computer simulation" OR "VR" OR "postural balance" OR "video games" OR "Wii" OR "Nintendo" OR "Virtual Realit*" OR "Exergaming" OR "Video Games" OR "Head Mounted Display" OR "Kinect" OR "Xbox" OR "Equine-Assisted Therapy" OR "Hippotherapy Simulator" OR "Equine Simulator" OR "Horseback" OR "game" OR "Games" OR "gaming" OR "Gamification" OR "Mobile Application" OR "mobile applications" OR "PlayStation" OR "Simulation Training" OR "Simulation technologies" OR "Simulation technology" OR "Patient Simulation" OR "virtual simulation" OR "Simulation" OR "Simulator" OR "VR-based" OR "Virtual reality based" AND ( LIMIT-TO ( PUBYEAR,2022) OR LIMIT-TO ( PUBYEAR,2021) OR LIMIT-TO ( PUBYEAR,2020) OR LIMIT-TO ( PUBYEAR,2019) OR LIMIT-TO ( PUBYEAR,2018) OR LIMIT-TO ( PUBYEAR,2017) OR LIMIT-TO ( PUBYEAR,2016) OR LIMIT-TO ( PUBYEAR,2015) OR LIMIT-TO ( PUBYEAR,2014) OR LIMIT-TO ( PUBYEAR,2013) OR LIMIT-TO ( PUBYEAR,2012) ) AND ( LIMIT-TO ( LANGUAGE,"English" ) ) |
| --- | --- |

The search was limited to 2012-2022 publication year and English language only

| Cochrane review  (Search were limited to 10 years and English only) | #1 | “Cervical” OR “neck” OR “back” OR “low back” OR “midthoracic” OR “thoracic” OR “lumbar” OR “sacrum” OR “sacral” OR “sacroiliac” OR “pelvic” OR “shoulder” OR “shoulder joint” OR “shoulder impingement syndrome” OR “subacromial” OR “hip” OR “hip osteoarthritis” OR “knee joint” OR “knee osteoarthritis” OR “knee” OR “osteoarthritis” OR “arthritis” OR “ankle” OR “ankle joint” OR “muscle” OR “muscle*“ OR “muscular” OR “bone” OR “bony” OR “joint” OR “joint*“ OR “spondylosis” OR “spondylolisthesis” OR “ankylosing” OR “spine” OR “spinal” OR “nociceptive” OR “low back pain” | |
| --- | --- | --- | --- |
|  | #2 | “Virtual Reality” OR “computer simulation” OR “VR” OR “postural balance” OR “video games” OR “Wii” OR “Nintendo” OR “Virtual Realit*“ OR “Exergaming” OR “Video Games” OR “Head Mounted Display” OR “Kinect” OR “Xbox” OR “Equine-Assisted Therapy” OR “Hippotherapy Simulator” OR “Equine Simulator” OR “Horseback” OR “game” OR “Games” OR “gaming” OR “Gamification” OR “Mobile Application” OR “mobile applications” OR “PlayStation” OR “Simulation Training” OR “Simulation technologies” OR “Simulation technology” OR “Patient Simulation” OR “virtual simulation” OR “Simulation” OR “Simulator” OR “VR-based” OR “Virtual reality-based” | |
|  | #3 | “Pain” OR “chronic Pain” OR “chronic” OR “acute” OR “disability” OR “ache” OR “discomfort” OR “Injury” OR “injuries” OR “injur*“ OR “spasm” OR “sprain” OR “strain” OR “tenderness” OR “contracture” | |
|  | #4 | | Combined 1#, 3# |
|  | #5 | | Combined #4 and #5 |

| CINHAL  Academic journal, English,  10 years 1/1/2012-31/12/2022 | AB ( “Cervical” OR “neck” OR “back” OR “low back” OR “midthoracic” OR “thoracic” OR “lumbar” OR “sacrum” OR “sacral” OR “sacroiliac” OR “pelvic” OR “shoulder” OR “shoulder joint” OR “shoulder impingement syndrome” OR “subacromial” OR “hip” OR “hip osteoarthritis” OR “knee joint” OR “knee osteoarthritis” OR “knee” OR “osteoarthritis” OR “arthritis” OR “ankle” OR “ankle joint” OR “muscle” OR “muscle*“ OR “muscular” OR “bone” OR “bony” OR “joint” OR “joint*“ OR “spondylosis” OR “spondylolisthesis” OR “ankylosing” OR “spine” OR “spinal” OR “nociceptive” OR “low back pain” ) AND AB ( “Pain” OR “chronic Pain” OR “chronic” OR “acute” OR “disability” OR “ache” OR “discomfort” OR “Injury” OR “injuries” OR “injur*“ OR “spasm” OR “sprain” OR “strain” OR “tenderness” OR “contracture” ) AND AB ( “Virtual Reality” OR “computer simulation” OR “VR” OR “postural balance” OR “video games” OR “Wii” OR “Nintendo” OR “Virtual Realit*“ OR “Exergaming” OR “Video Games” OR “Head Mounted Display” OR “Kinect” OR “Xbox” OR “Equine-Assisted Therapy” OR “Hippotherapy Simulator” OR “Equine Simulator” OR “Horseback” OR “game” OR “Games” OR “gaming” OR “Gamification” OR “Mobile Application” OR “mobile applications” OR “PlayStation” OR “Simulation Training” OR “Simulation technologies” OR “Simulation technology” OR “Patient Simulation” OR “virtual simulation” OR “Simulation” OR “Simulator” OR “VR-based” OR “Virtual reality-based” ) |
| --- | --- |

| Ovid (MEDLINE)  AMED  EMBASE | #1 | Cervical.mp. or Neck Muscles/ or Neck/ or *Neck Pain/ or neck.mp. or Neck Injuries/ or Back Muscles/ or Back/ or *Back Pain/ or back.mp. or *Low Back Pain/ or Back Injuries/ or *Chronic Pain/ or *Low Back Pain/ or *Back Pain/ or low back.mp. or midthoracic.mp. or thoracic.mp. or lumbar.mp. or sacrum.mp. or Sacrum/ or sacral.mp. or *Low Back Pain/ or Sacroiliac Joint/ or sacroiliac.mp. or Spondylitis, Ankylosing/ or pelvic.mp. or Pelvic Girdle Pain/ or Pelvic Pain/ or Shoulder Injuries/ or Shoulder Pain/ or Shoulder Joint/ or shoulder.mp. or Shoulder Impingement Syndrome/ or shoulder joint.mp. or Shoulder Joint/ or shoulder impingement syndrome.mp. or Rotator Cuff/ or Shoulder Impingement Syndrome/ or Hip Injuries/ or hip.mp. or Osteoarthritis, Hip/ or Hip/ or Hip Contracture/ or hip osteoarthritis.mp. or Osteoarthritis, Hip/ or knee joint.mp. or Knee Joint/ or knee osteoarthritis.mp. or Osteoarthritis, Knee/ or Knee/ or Knee Injuries/ or knee.mp. or Knee Prosthesis/ or osteoarthritis.mp. or Osteoarthritis, Hip/ or Osteoarthritis/ or Osteoarthritis, Spine/ or Osteoarthritis, Knee/ or Arthritis/ or Arthritis, Rheumatoid/ or arthritis.mp. or ankle.mp. or Ankle Joint/ or Ankle/ or Ankle Injuries/ or ankle joint.mp. or Ankle Joint/ or muscle.mp. or Muscles/ or muscle*.mp. or muscular.mp. or bone.mp. or "Bone and Bones"/ or bony.mp. or joint.mp. or Joints/ or joint*.mp. or spondylosis.mp. or Spondylosis/ or spondylolisthesis.mp. or Spondylolisthesis/ or Spondylitis, Ankylosing/ or ankylosing.mp. or Spine/ or spine.mp. or Muscular Atrophy, Spinal/ or Spinal Injuries/ or spinal.mp. or Nociceptors/ or nociceptive.mp. or Pain/ or Nociceptive Pain/ or low back pain.mp. or Back Pain/ or exp *Low Back Pain/ or |
| --- | --- | --- |
|  | #2 | Virtual Reality.mp. or exp *Virtual Reality/ or computer simulation.mp. or Computer Simulation/ or Virtual Reality/ or VR.mp. or Computer Simulation/ or postural balance.mp. or Postural Balance/ or video games.mp. or Video Games/ or Wii.mp. or Nintendo.mp. Virtual Realit*.mp. or Virtual Reality Exposure Therapy/ or Exergaming.mp. or Exergaming/ or Head Mounted Display.mp. or Kinect.mp. or Video Games/ or Xbox.mp. or Virtual Reality/ or Equine-Assisted Therapy.mp. or Equine-Assisted Therapy or Equine Simulator.mp. or Horseback.mp. or Equine-Assisted Therapy/ or game.mp. or Video Games/ or Games.mp. or gaming.mp. or Gamification.mp. or Gamification/ or Mobile Application.mp. or Mobile Applications/ or mobile applications.mp. or Mobile Applications/ or PlayStation.mp. or PlayStation.mp. or Simulation Training.mp. or Simulation Training/ or Patient Simulation/ or Computer Simulation/ or Simulation Training/ or Simulation technologies.mp. or Simulation Training/ or Simulation technologies.mp. or Simulation technology.mp. Patient Simulation.mp. or Patient Simulation/ or virtual simulation.mp. or Simulation.mp. or Simulator.mp. or VR-based.mp. or Virtual reality-based.mp. |
|  | #3 | Pain.mp. or Pain/ or chronic Pain.mp. or Chronic Pain/ or chronic.mp. or acute.mp. or disability.mp. or ache.mp. or Pain/ or discomfort.mp. or Injury.mp. or injuries.mp. or injur*.mp. or spasm.mp. or Spasm/ or strain.mp. or "Sprains and Strains"/ or tenderness.mp. or Contracture/ or contracture.mp. |
|  | #4 | #1 AND #2 AND #3 |
|  | #5 | limit 86 to (english language and yr="2012 -Current") |
|  | #6 | limit 87 to "reviews (best balance of sensitivity and specificity)" |

| Web of Science  The search was refined to English only and review articles | #1 | ((TI=(“Cervical” OR “neck” OR “back” OR “low back” OR “midthoracic” OR “thoracic” OR “lumbar” OR “sacrum” OR “sacral” OR “sacroiliac” OR “pelvic” OR “shoulder” OR “shoulder joint” OR “shoulder impingement syndrome” OR “subacromial” OR “hip” OR “hip osteoarthritis” OR “knee joint” OR “knee osteoarthritis” OR “knee” OR “osteoarthritis” OR “arthritis” OR “ankle” OR “ankle joint” OR “muscle” OR “muscle*“ OR “muscular” OR “bone” OR “bony” OR “joint” OR “joint*“ OR “spondylosis” OR “spondylolisthesis” OR “ankylosing” OR “spine” OR “spinal” OR “nociceptive” OR “low back pain”)) OR AB=(“Cervical” OR “neck” OR “back” OR “low back” OR “midthoracic” OR “thoracic” OR “lumbar” OR “sacrum” OR “sacral” OR “sacroiliac” OR “pelvic” OR “shoulder” OR “shoulder joint” OR “shoulder impingement syndrome” OR “subacromial” OR “hip” OR “hip osteoarthritis” OR “knee joint” OR “knee osteoarthritis” OR “knee” OR “osteoarthritis” OR “arthritis” OR “ankle” OR “ankle joint” OR “muscle” OR “muscle*“ OR “muscular” OR “bone” OR “bony” OR “joint” OR “joint*“ OR “spondylosis” OR “spondylolisthesis” OR “ankylosing” OR “spine” OR “spinal” OR “nociceptive” OR “low back pain”)) OR KP=(“Cervical” OR “neck” OR “back” OR “low back” OR “midthoracic” OR “thoracic” OR “lumbar” OR “sacrum” OR “sacral” OR “sacroiliac” OR “pelvic” OR “shoulder” OR “shoulder joint” OR “shoulder impingement syndrome” OR “subacromial” OR “hip” OR “hip osteoarthritis” OR “knee joint” OR “knee osteoarthritis” OR “knee” OR “osteoarthritis” OR “arthritis” OR “ankle” OR “ankle joint” OR “muscle” OR “muscle*“ OR “muscular” OR “bone” OR “bony” OR “joint” OR “joint*“ OR “spondylosis” OR “spondylolisthesis” OR “ankylosing” OR “spine” OR “spinal” OR “nociceptive” OR “low back pain”) |
| --- | --- | --- |
|  | #2 | ((TI=(“Virtual Reality” OR “computer simulation” OR “VR” OR “postural balance” OR “video games” OR “Wii” OR “Nintendo” OR “Virtual Realit*“ OR “Exergaming” OR “Video Games” OR “Head Mounted Display” OR “Kinect” OR “Xbox” OR “Equine-Assisted Therapy” OR “Hippotherapy Simulator” OR “Equine Simulator” OR “Horseback” OR “game” OR “Games” OR “gaming” OR “Gamification” OR “Mobile Application” OR “mobile applications” OR “PlayStation” OR “Simulation Training” OR “Simulation technologies” OR “Simulation technology” OR “Patient Simulation” OR “virtual simulation” OR “Simulation” OR “Simulator” OR “VR-based” OR “Virtual reality-based”)) OR AB=(“Virtual Reality” OR “computer simulation” OR “VR” OR “postural balance” OR “video games” OR “Wii” OR “Nintendo” OR “Virtual Realit*“ OR “Exergaming” OR “Video Games” OR “Head Mounted Display” OR “Kinect” OR “Xbox” OR “Equine-Assisted Therapy” OR “Hippotherapy Simulator” OR “Equine Simulator” OR “Horseback” OR “game” OR “Games” OR “gaming” OR “Gamification” OR “Mobile Application” OR “mobile applications” OR “PlayStation” OR “Simulation Training” OR “Simulation technologies” OR “Simulation technology” OR “Patient Simulation” OR “virtual simulation” OR “Simulation” OR “Simulator” OR “VR-based” OR “Virtual reality-based”)) OR KP=(“Virtual Reality” OR “computer simulation” OR “VR” OR “postural balance” OR “video games” OR “Wii” OR “Nintendo” OR “Virtual Realit*“ OR “Exergaming” OR “Video Games” OR “Head Mounted Display” OR “Kinect” OR “Xbox” OR “Equine-Assisted Therapy” OR “Hippotherapy Simulator” OR “Equine Simulator” OR “Horseback” OR “game” OR “Games” OR “gaming” OR “Gamification” OR “Mobile Application” OR “mobile applications” OR “PlayStation” OR “Simulation Training” OR “Simulation technologies” OR “Simulation technology” OR “Patient Simulation” OR “virtual simulation” OR “Simulation” OR “Simulator” OR “VR-based” OR “Virtual reality-based”) |
|  | #3 | ((TI=(“Pain” OR “chronic Pain” OR “chronic” OR “acute” OR “disability” OR “ache” OR “discomfort” OR “Injury” OR “injuries” OR “injur*“ OR “spasm” OR “sprain” OR “strain” OR “tenderness” OR “contracture”)) OR AB=(“Pain” OR “chronic Pain” OR “chronic” OR “acute” OR “disability” OR “ache” OR “discomfort” OR “Injury” OR “injuries” OR “injur*“ OR “spasm” OR “sprain” OR “strain” OR “tenderness” OR “contracture”)) OR KP=(“Pain” OR “chronic Pain” OR “chronic” OR “acute” OR “disability” OR “ache” OR “discomfort” OR “Injury” OR “injuries” OR “injur*“ OR “spasm” OR “sprain” OR “strain” OR “tenderness” OR “contracture”) |
|  | #4 | #1 AND 2# AND #3 |
|  | #5 | Publication date from 2012-1-1 to 2022-04-10 |

Updated search terms for the search conducted on **3 July 2024**

| Ovid |  |  |
| --- | --- | --- |
|  | 1 | Cervical.mp. or Neck Muscles/ or Neck/ or *Neck Pain/ or neck.mp. or Neck Injuries/ or Back Muscles/ or Back/ or *Back Pain/ or back.mp. or *Low Back Pain/ or Back Injuries/ or *Chronic Pain/ or *Low Back Pain/ or *Back Pain/ or low back.mp. or midthoracic.mp. or thoracic.mp. or lumbar.mp. or sacrum.mp. or Sacrum/ or sacral.mp. or *Low Back Pain/ or Sacroiliac Joint/ or sacroiliac.mp. or Spondylitis, Ankylosing/ or pelvic.mp. or Pelvic Girdle Pain/ or Pelvic Pain/ or Shoulder Injuries/ or Shoulder Pain/ or Shoulder Joint/ or shoulder.mp. or Shoulder Impingement Syndrome/ or shoulder joint.mp. or Shoulder Joint/ or shoulder impingement syndrome.mp. or Rotator Cuff/ or Shoulder Impingement Syndrome/ or Hip Injuries/ or hip.mp. or Osteoarthritis, Hip/ or Hip/ or Hip Contracture/ or hip osteoarthritis.mp. or Osteoarthritis, Hip/ or knee joint.mp. or Knee Joint/ or knee osteoarthritis.mp. or Osteoarthritis, Knee/ or Knee/ or Knee Injuries/ or knee.mp. or Knee Prosthesis/ or osteoarthritis.mp. or Osteoarthritis, Hip/ or Osteoarthritis/ or Osteoarthritis, Spine/ or Osteoarthritis, Knee/ or Arthritis/ or Arthritis, Rheumatoid/ or arthritis.mp. or ankle.mp. or Ankle Joint/ or Ankle/ or Ankle Injuries/ or ankle joint.mp. or Ankle Joint/ or muscle.mp. or Muscles/ or muscle*.mp. or muscular.mp. or bone.mp. or "Bone and Bones"/ or bony.mp. or joint.mp. or Joints/ or joint*.mp. or spondylosis.mp. or Spondylosis/ or spondylolisthesis.mp. or Spondylolisthesis/ or Spondylitis, Ankylosing/ or ankylosing.mp. or Spine/ or spine.mp. or Muscular Atrophy, Spinal/ or Spinal Injuries/ or spinal.mp. or Nociceptors/ or nociceptive.mp. or Pain/ or Nociceptive Pain/ or low back pain.mp. or Back Pain/ or exp *Low Back Pain/ |
|  | 2 | Virtual Reality.mp. or exp *Virtual Reality/ or computer simulation.mp. or Computer Simulation/ or Virtual Reality/ or VR.mp. or Computer Simulation/ or postural balance.mp. or Postural Balance/ or video games.mp. or Video Games/ or Wii.mp. or Nintendo.mp. or Virtual Reality Exposure Therapy/ or Exergaming.mp. or Exergaming/ or Head Mounted Display.mp. or Kinect.mp. or Video Games/ or Xbox.mp. or Virtual Reality/ or Equine-Assisted Therapy.mp. or Equine-Assisted Therapy.mp. or Equine Simulator.mp. or Horseback.mp. or Equine-Assisted Therapy/ or game.mp. or Video Games/ or Games.mp. or gaming.mp. or Gamification.mp. or Gamification/ or Mobile Application.mp. or Mobile Applications/ or mobile applications.mp. or Mobile Applications/ or PlayStation.mp. or PlayStation.mp. or Simulation Training.mp. or Simulation Training/ or Patient Simulation/ or Computer Simulation/ or Simulation Training/ or Simulation technologies.mp. or Simulation Training/ or Simulation technologies.mp. or Simulation technology.mp. or Patient Simulation/ or virtual simulation.mp. or Simulation.mp. or Simulator.mp. or VR-based.mp. or Virtual reality-based.mp. [mp=ab, hw, ti, tn, ot, dm, mf, dv, kf, fx, dq, bt, nm, ox, px, rx, an, ui, sy, ux, mx, tx, sh, ct] |
|  | 3 | Pain.mp. or Pain/ or chronic Pain.mp. or Chronic Pain/ or chronic.mp. or acute.mp. or disability.mp. or ache.mp. or Pain/ or discomfort.mp. or Injury.mp. or injuries.mp. or injur*.mp. or spasm.mp. or Spasm/ or strain.mp. or "Sprains and Strains"/ or tenderness.mp. or Contracture/ or contracture.mp. |
|  | 4 | limit 3 to english language [Limit not valid in MWIC,Zoological Record,Books@Ovid,Journals@Ovid,Your Journals@Ovid,HMIC; records were retained] |
|  | 5 | limit 4 to yr="2022 - 2024" |
|  | 6 | limit 5 to "systematic review" [Limit not valid in AMED,FSTA,INSPEC,IPAB,Zoological Record,Books@Ovid,Journals@Ovid,Your Journals@Ovid,HMIC; records were retained] |
|  | 7 | 1 and 2 and 3 and 4 and 5 and 6 |

Scopus

|  |  | "Pain" OR "chronic Pain" OR "chronic" OR "acute" OR "disability" OR "ache" OR "Injury" OR "injuries" OR "injur*" OR "spasm" OR "sprain" OR "strain" OR "tenderness" OR "contracture" OR "Cervical" OR "neck" OR "back" OR "low back" OR "lumbar" OR "sacrum" OR "sacral" OR "sacroiliac" OR "pelvic" OR "shoulder" OR "shoulder joint" OR "shoulder impingement syndrome" OR "subacromial" OR "hip" OR "hip osteoarthritis" OR "knee joint" OR "knee osteoarthritis" OR "knee" OR "osteoarthritis" OR "arthritis" OR "ankle" OR "ankle joint" OR "muscle" OR "muscular" OR "bone" OR "bony" OR "joint" " OR " spine " OR " nociceptive " OR " back AND pain |
| --- | --- | --- |
|  |  | "Virtual Reality" OR "computer simulation" OR "postural balance" OR "video games" OR "Wii" OR "Nintendo" OR "Virtual Realit*" OR "Video Games" OR "Head Mounted Display" OR "Equine-Assisted Therapy" OR "game" OR "Games" OR "gaming" OR "Mobile Application" OR "mobile applications" OR "PlayStation" OR "Simulation Training" OR "Simulation technologies" OR "Simulation technology" OR "Patient Simulation" OR "virtual simulation" OR "Simulation" OR "Virtual reality based" |
|  |  | From 2022 to 2024 |
|  |  | Limited to Review |
|  |  | Limited to English |

Web of Science

|  |  | “Cervical” OR “neck” OR “back” OR “low back” OR “midthoracic” OR “thoracic” OR “lumbar” OR “sacrum” OR “sacral” OR “sacroiliac” OR “pelvic” OR “shoulder” OR “shoulder joint” OR “shoulder impingement syndrome” OR “subacromial” OR “hip” OR “hip osteoarthritis” OR “knee joint” OR “knee osteoarthritis” OR “knee” OR “osteoarthritis” OR “arthritis” OR “ankle” OR “ankle joint” OR “muscle” OR “muscle*“ OR “muscular” OR “bone” OR “bony” OR “joint” OR “joint*“ OR “spondylosis” OR “spondylolisthesis” OR “ankylosing” OR “spine” OR “spinal” OR “nociceptive” OR “low back pain” (All Fields) |
| --- | --- | --- |
|  |  | “Virtual Reality” OR “computer simulation” OR “VR” OR “postural balance” OR “video games” OR “Wii” OR “Nintendo” OR “Virtual Realit*“ OR “Exergaming” OR “Video Games” OR “Head Mounted Display” OR “Kinect” OR “Xbox” OR “Equine-Assisted Therapy” OR “Hippotherapy Simulator” OR “Equine Simulator” OR “Horseback” OR “game” OR “Games” OR “gaming” OR “Gamification” OR “Mobile Application” OR “mobile applications” OR “PlayStation” OR “Simulation Training” OR “Simulation technologies” OR “Simulation technology” OR “Patient Simulation” OR “virtual simulation” OR “Simulation” OR “Simulator” OR “VR-based” OR “Virtual reality-based” (All Fields) |
|  |  | “Pain” OR “chronic Pain” OR “chronic” OR “acute” OR “disability” OR “ache” OR “discomfort” OR “Injury” OR “injuries” OR “injur*“ OR “spasm” OR “sprain” OR “strain” OR “tenderness” OR “contracture” (All Fields) |
|  |  | Refined By: Publication Years: 2024 or 2023 or 2022 AND Document Types: Review Article AND Languages: English |

CINAHL Ultimate

|  |  | “Cervical” OR “neck” OR “back” OR “low back” OR “midthoracic” OR “thoracic” OR “lumbar” OR “sacrum” OR “sacral” OR “sacroiliac” OR “pelvic” OR “shoulder” OR “shoulder joint” OR “shoulder impingement syndrome” OR “subacromial” OR “hip” OR “hip osteoarthritis” OR “knee joint” OR “knee osteoarthritis” OR “knee” OR “osteoarthritis” OR “arthritis” OR “ankle” OR “ankle joint” OR “muscle” OR “muscle*“ OR “muscular” OR “bone” OR “bony” OR “joint” OR “joint*“ OR “spondylosis” OR “spondylolisthesis” OR “ankylosing” OR “spine” OR “spinal” OR “nociceptive” OR “low back pain” |
| --- | --- | --- |
|  |  | “Virtual Reality” OR “computer simulation” OR “VR” OR “postural balance” OR “video games” OR “Wii” OR “Nintendo” OR “Virtual Realit*“ OR “Exergaming” OR “Video Games” OR “Head Mounted Display” OR “Kinect” OR “Xbox” OR “Equine-Assisted Therapy” OR “Hippotherapy Simulator” OR “Equine Simulator” OR “Horseback” OR “game” OR “Games” OR “gaming” OR “Gamification” OR “Mobile Application” OR “mobile applications” OR “PlayStation” OR “Simulation Training” OR “Simulation technologies” OR “Simulation technology” OR “Patient Simulation” OR “virtual simulation” OR “Simulation” OR “Simulator” OR “VR-based” OR “Virtual reality-based” |
|  |  | “Pain” OR “chronic Pain” OR “chronic” OR “acute” OR “disability” OR “ache” OR “discomfort” OR “Injury” OR “injuries” OR “injur*“ OR “spasm” OR “sprain” OR “strain” OR “tenderness” OR “contracture” |
|  |  | Limiters - Publication Date: 20220101-20241231; Clinical Queries: Review - Best Balance; Language: English; Publication Type: Academic Journal; Age Groups: All Adult |
|  |  | Source Types: Academic Journals |
|  |  | Language: English |
|  |  | Publication Date: From 2022-01-01to 2024-12-31 |

| Cochrane review  (Search were limited to 10 years and English only) | #1 | (Cervical) OR (neck) OR (back) OR (low NEXT back) OR (mid NEXT thoracic) OR (thoracic) OR (lumbar) OR (sacrum) OR (sacral) OR (sacroiliac) OR (pelvic) OR (shoulder) OR (shoulder NEXT joint) OR (shoulder impingement syndrome) OR (subacromial) OR (hip) OR (hip NEXT osteoarthritis) OR (knee joint) OR (knee osteoarthritis) OR (knee) OR (osteoarthritis) OR (arthritis) OR (ankle) OR (ankle NEXT joint) OR (muscle) OR (muscle*) OR (muscular) OR (bone) OR (bony) OR (joint) OR (joint*) OR (spondylosis) OR (spondylolisthesis) OR (ankylosing) OR (spine) OR (spinal) OR (nociceptive) OR (low NEXT back NEXT pain) | |
| --- | --- | --- | --- |
|  | #2 | (Virtual NEXT Reality) OR (computer NEXT simulation) OR (VR) OR (postural NEXT balance) OR (video NEXT games) OR (Wii) OR (Nintendo) OR (Virtual NEXT Realit*) OR (Exergaming) OR (Video NEXT Games) OR (Head NEXT Mounted NEXT Display) OR (Kinect) OR (Xbox) OR (Equine-Assisted NEXT Therapy) OR (Hippotherapy NEXT Simulator) OR (Equine NEXT Simulator) OR (Horseback) OR (game) OR (Games) OR (gaming) OR (Gamification) OR (Mobile NEXT Application) OR (mobile NEXT applications) OR (PlayStation) OR (Simulation NEXT Training) OR (Simulation NEXT technologies) OR (Simulation NEXT technology) OR (Patient NEXT Simulation) OR (virtual NEXT simulation) OR (Simulation) OR (Simulator) OR (VR-based) OR (Virtual NEXT reality-based) | |
|  | #3 | (Pain) OR (chronic Pain) OR (chronic) OR (acute) OR (disability) OR (ache) OR (discomfort) OR (Injury) OR (injuries) OR (injur*) OR (spasm) OR (sprain) OR (strain) OR (tenderness) OR (contracture) | |
|  | #4 | | #1 AND #3 |
|  | #5 | | #2 AND #4 |
