# Supplementary material for: Virtual Reality for Patients With Chronic Musculoskeletal Pain and Disability: An Umbrella Review of Systematic Reviews
Source: Health Sci Rep. 2025 Aug 12;8(8):e71163. doi: 10.1002/hsr2.71163 (PMC12343317; doi:10.1002/hsr2.71163)
Supplement: Supplementary file 2 — S2 File. Supplemental Methods and Results. [file HSR2-8-e71163-s006.docx]

# Supplemental Methods

## Data extraction

The data extraction form included authors, conditions, number of studies included, participants (i.e., characteristics and total number), settings, type of VR platform used for intervention, list of interventions with VR, list of comparator group interventions, VR intervention time, duration of rehabilitation, types of studies included, country of origin of included studies, appraisal instruments used, appraisal rating, method of analysis, outcome assessed, measurement instrument, results, and heterogeneity.

## Quality assessment

The AMSTAR-2 is valid and moderately reliable for critically appraising SRs that include both randomised and non-randomised studies [1]. The AMSTAR-2 tool has 16 domains, seven of which are critical for high-quality SRs: registration of the protocol (item 2), a satisfactory literature search (item 4), reasons for excluding particular publications (item 7), assessment of the risk of bias (item 9), meta-analytical approach’s suitability (item 11), taking into account the risk of bias when interpreting the results (item 13), and evaluation of the existence and potential effect of publication bias (item 15). The non-critical domains are the presence of the PICO format (item 1), justification of study designs of the primary studies (item 3), selection of the primary studies (item 5), data extraction performed by two reviewers (item 6), an adequate description of studies that were included (item 8), reporting fund sources of included studies (item 10), evaluation of the influence of the risk of bias on SR results (item 12), a satisfactory explanation for heterogeneity (14), and reporting any conflict of interest (16). The quality of eligible studies was classified as high (no or one non-critical weakness), moderate (more than one non-critical weakness), low (one critical flaw with or without non-critical weaknesses), or critically low (more than one critical flaw with or without non-critical weaknesses) [2].

The certainty in the body of evidence was assessed using the GRADE [3]. The body of evidence was downgraded one level (e.g., from moderate to low) based on each of five factors: (1) risk of bias (downgraded if equal to or more than 25% of the included primary studies are at some concerns or high risk of bias on at least one item across all risk of bias criteria); (2) inconsistency (downgraded if the heterogeneity is I^2^ ≥ 50%); (3) indirectness (downgraded when 50% of the participants were outside the target group); (4) imprecision (downgraded if the sample size < 400 for each outcome); and (5) evidence of publication bias (downgraded when the reported funnel plot is asymmetrical, and the recommended number for assessing publication bias (10 primary studies “[4]”) met) [5]. The assessment started from high and was only downgraded. No upgrade was performed. This review's GRADE assessment relied on the data collected from the SR synthesis.

## Data synthesis

The tables included authors, year, participants number, setting, country of the included studies, appraisal tool, appraisal rating, range years of the included studies, conditions, type of VR intervention, list of interventions of the comparator group, duration of the session, frequency, rehabilitation duration, results, effect estimated size, level of evidence, and heterogeneity. The rating assessment, including the overall confidence and certainty in the body of evidence in the results of the SRs, are reported in the provided tables.

# Supplemental Results

## Virtual reality group

Immersive VR was utilised for patients with CNP (n = 5) and CLBP (n = 6). In addition, non-immersive VR was utilised for patients with CNP (n = 2) and CLBP (n = 23). All the studies included VR interventions, either combined with other interventions (n = 18) or standalone interventions (n = 18). The VR intervention was in addition to physiotherapy (n = 8), exercises (n = 1), care as usual (n = 1), lumbar strengthening exercises (n = 1), physical activity (n = 1), core exercises and strengthening training (n = 1), magnetic therapy (n = 1), kinematic training (n = 1), motor control exercises (n = 1), sensorimotor training (n = 1), and psychoeducation (n = 1).

## Control group

Control groups received various types of interventions, including conventional balance training + physiotherapy (n = 6), care as usual (n = 5), daily life routines (n = 3), kinematic training using a head-mounted laser (n = 2), conventional proprioception training (n = 1), motor control exercises (n = 1), general sensorimotor training + care as usual (n = 1), trunk-stabilising exercises + physiotherapy (n = 1), physiotherapy (n = 1), core exercises and strength training (n = 1), lumbar strengthening exercise (n = 1), stabilisation exercise (n = 1), watching video (n = 1), traditional training (n = 1), sham VR (n = 1), oral treatment (n = 1), conventional thermal magnetic therapy (n = 1), audio instruction programme (n = 1), back strengthening exercise + physiotherapy (n = 1), chair-based exercises + psychoeducation (n = 1) and no intervention (n = 3).

## Countries and settings of included primary studies

Non-immersive VR was delivered to patients with CNP in four countries, including Australia (n = 2), Turkey (n = 1), Germany (n = 1), and Sweden (n = 1). Immersive VR was delivered to patients with CLBP in five countries, including Germany (n = 1), the United States (n = 2), Iran (n = 1), Finland (n = 1), and the Netherlands (n = 1). However, non-immersive VR was utilised for patients with CNP in Iran (n = 1) and Spain (n = 1), whereas for patients with CLBP in South Korea (n = 7), Australia (n = 1), Saudi Arabia (n = 6), Turkey (n = 2), United States (n = 1), Japan (n = 2), China (n = 1), Belgium (n = 1), Pakistan (n = 1) and Switzerland (n = 1).

## Outcomes

## Primary outcomes

### Pain

The following measures were utilised to assess pain outcomes: visual analogue scale [6-11], 11-point numerical pain rating scale [6, 8, 9, 11], Defense and Veterans Pain Rating Scale [6, 9, 11], present pain intensity scale [7]. Kumar [12] did not specify the outcome measure.

### Disability

Disability was measured using the Neck Disability Index [7, 8, 10], Oswestry Disability Index (ODI) [6, 7, 9], modified ODI [11], and Roland–Morris Disability Questionnaire [6, 9, 11], and Functional Ability Questionnaire for Measuring Back Pain–Related Disability [11].

### Secondary outcomes

#### Kinesobphia

Kinesiophobia was evaluated using the Fear-Avoidance Beliefs Questionnaire [9], Tampa Scale for Kinesiophobia (TSK) [7-9], TSK-17 [6, 10, 11] and TSK-11 [11].

# References

1. Lorenz RC, Matthias K, Pieper D, Wegewitz U, Morche J, Nocon M, et al. A psychometric study found AMSTAR 2 to be a valid and moderately reliable appraisal tool. J Clin Epidemiol. 2019;114:133-40.

2. Shea BJ, Reeves BC, Wells G, Thuku M, Hamel C, Moran J, et al. AMSTAR 2: a critical appraisal tool for systematic reviews that include randomised or non-randomised studies of healthcare interventions, or both. BMJ. 2017;358:j4008.

3. Balshem H, Helfand M, Schünemann HJ, Oxman AD, Kunz R, Brozek J, et al. GRADE guidelines: 3. Rating the quality of evidence. J Clin Epidemiol. 2011;64:401-6.

4. Guyatt GH, Oxman AD, Montori V, Vist G, Kunz R, Brozek J, et al. GRADE guidelines: 5. Rating the quality of evidence—publication bias. J Clin Epidemiol. 2011;64:1277-82.

5. Ahern MM, Dean LV, Stoddard CC, Agrawal A, Kim K, Cook CE, et al. The effectiveness of virtual reality in patients with spinal pain: a systematic review and meta-analysis. Pain Pract. 2020;20:656-75.

6. Brea-Gómez B, Torres-Sánchez I, Ortiz-Rubio A, Calvache-Mateo A, Cabrera-Martos I, López-López L, et al. Virtual reality in the treatment of adults with chronic low back pain: a systematic review and meta-analysis of randomized clinical trials. Int J Environ Res Public Health. 2021;18:11806.

7. Grassini S. Virtual reality assisted non-pharmacological treatments in chronic pain management: a systematic review and quantitative meta-analysis. Int J Environ Res Public Health. 2022;19:4071.

8. Hao J, He Z, Chen Z, Remis A. Virtual reality training versus conventional rehabilitation for chronic neck pain: a systematic review and meta-analysis. PM&R. 20241-11.

9. Li R, Li Y, Kong Y, Li H, Hu D, Fu C, et al. Virtual reality-based training in chronic low back pain: systematic review and meta-analysis of randomized controlled trials. J Med Internet Res. 2024;26:e45406.

10. Ye G, Koh RGL, Jaiswal K, Soomal H, Kumbhare D. The use of virtual reality in the rehabilitation of chronic nonspecific neck pain: a systematic review and meta-analysis. Clin J Pain. 2023;39:491-500.

11. Zhang TT, Li X, Zhou X, Zhan LX, Wu F, Huang ZF, et al. Virtual reality therapy for the management of chronic spinal pain: systematic review and meta-analysis. JMIR Serious Games 2024;12.

12. Kumar V, Vatkar AJ, Kataria M, Dhatt SS, Baburaj V. Virtual reality is effective in the management of chronic low back ache in adults: a systematic review and meta-analysis of randomized controlled trials. Eur Spine J. 2024;33:474-80.
