# Supplementary material for: Virtual Reality for Patients With Chronic Musculoskeletal Pain and Disability: An Umbrella Review of Systematic Reviews
Source: Health Sci Rep. 2025 Aug 12;8(8):e71163. doi: 10.1002/hsr2.71163 (PMC12343317; doi:10.1002/hsr2.71163)
Supplement: Supplementary file 3 — S3 File. Risk of Bias and Quality Assessment as Reported by Systematic Reviews. [file HSR2-8-e71163-s008.docx]

Table 1: Risk of Bias According to the Included Systematic Reviews’ Reports

| Authors | Appraisal tool | Appraisal rating |
| --- | --- | --- |
| Brea-Gómez et al. (2021) | The Cochrane Collaboration’s tool for assessing risk of bias in randomised trials | 1. Random sequence generation (selection bias): four studies had an unclear risk of bias, and 10 had a low risk of bias. 2. Allocation concealment (selection bias): 11 studies had an unclear risk of bias, and three had a low risk of bias. 3. Blinding of participants and personnel (performance bias): four studies had a high risk of bias, five had an unclear risk of bias, and five had a low risk of bias. 4. Blinding of outcome assessment (detection bias): two studies had a high risk of bias, seven had an unclear risk of bias, and five had a low risk of bias. 5. Incomplete outcome data (attrition bias): two studies had an unclear risk of bias, and 12 had a low risk of bias. 6. Selective reporting (reporting bias): 11 studies had an unclear risk of bias, and three had low risk of bias. 7. Other sources of bias: all 14 studies had an unclear risk of bias. |
| Grassini (2022) | A Cochrane risk of bias assessment tool: for non-randomized studies of interventions (ACROBATNRSI) | 1. Random sequence generation (selection bias): all studies had a low risk of bias. 2. Allocation concealment (selection bias): all studies had a low risk of bias. 3. Blinding of participants and personnel (performance bias): three studies had a high risk of bias, and one had an unclear risk of bias. 4. Blinding of outcome assessment (detection bias): two studies had a high risk of bias, and two had an unclear risk of bias. 5. Incomplete outcome data (attrition bias): one study had an unclear risk of bias, and six had a low risk of bias. 6. Selective reporting (reporting bias): all studies had a low risk of bias. 7. Other sources of bias: All studies had an unclear risk of bias. |
| Hao et al. (2024) | Physiotherapy Evidence Database scale | 1. Eligibility criteria specified: all studies had a Yes. 2. Random sequence generation: all studies had a low risk of bias. 3. Allocation concealment: four studies had a high risk of bias, and two had a low risk of bias. 4. Group similarity at baseline: all studies had a low risk of bias. 5. Blinding of participants: all studies had a high risk of bias. 6. Blinding of therapists: all studies had a high risk of bias. 7. Blinding of assessors: three studies had a high risk of bias, and three had a low risk of bias. 8. Incomplete outcome data: all studies had a low risk of bias. 9. Intention to treat (attrition bias): four studies had a high risk of bias, and two had a low risk of bias. 10. Between-group comparison: all studies had a low risk of bias. 11. Point estimates and variability: all studies had a low risk of bias. |
| Kumar et al. (2024) | Cochrane risk-of-bias tool | 1. Random sequence generation (selection bias): all studies had a low risk of bias. 2. Allocation concealment (selection bias): two studies had a high risk of bias, two had an unclear risk of bias, and three had a low risk of bias. 3. Blinding of participants and personnel (performance bias): two studies had a high risk of bias, two had an unclear risk of bias, and three had a low risk of bias. 4. Blinding of outcome assessment (detection bias): two studies had a high risk of bias, one had an unclear risk of bias, and four had a low risk of bias. 5. Incomplete outcome data (attrition bias): all studies had a low risk of bias. 6. Selective reporting (reporting bias): all studies had a low risk of bias. 7. Other sources of bias: all studies had a low risk of bias. |
| Li et al. (2024) | A revised tool for assessing risk of bias in randomised trials (RoB 2) tool | 1. Randomisation process: six studies had a some concerns risk of bias, and 14 had a low risk of bias. 2. Deviations from the intended interventions: 15 studies had a some concerns risk of bias, and five had a low risk of bias. 3. Missing outcome data: six studies had a some concerns risk of bias, and 14 had a low risk of bias. 4. Measurement of the outcome: two studies had a high risk of bias, four had a some concerns risk of bias, and 14 had a low risk of bias. 5. Selection of the reported results: seven studies had a some concerns risk of bias, and 13 had a low risk of bias.   Overall bias: two studies had a high risk of bias, 17 had a some concerns risk of bias, and one had a low risk of bias. |
| Ye et al. (2023) | A revised tool for assessing risk of bias in randomised trials (RoB 2) tool | 1. Randomisation process: four studies had a low risk of bias, and one had a some concerns risk of bias. 2. Deviations from the intended interventions: four studies had a low risk of bias, and one had a high risk of bias. 3. Missing outcome data: all studies had a low risk of bias. 4. Measurement of the outcome: four studies had a low risk of bias, and one had a some concerns risk of bias. 5. Selection of the reported results: all studies had a low risk of bias.   Overall bias: four studies had a low risk of bias, and one had a high risk of bias. |
| Zhang et al. (2024) | Cochrane Risk of Bias tool | 1. Random sequence generation (selection bias): one study had an unclear risk of bias, and 15 had a low risk of bias. 2. Allocation concealment (selection bias): eleven studies had an unclear risk of bias, and five had a low risk of bias. 3. Blinding of participants and personnel (performance bias): ten studies had a high risk of bias, three had an unclear risk of bias, and one had a low risk of bias. 4. Blinding of outcome assessment (detection bias): four studies had a high risk of bias, three had an unclear risk of bias, and nine had a low risk of bias. 5. Incomplete outcome data (attrition bias): all studies had a low risk of bias. 6. Selective reporting (reporting bias): all studies had a low risk of bias. 7. Other sources of bias: all studies had a low risk of bias. |

Table 2: Quality Assessment According to the Included Systematic Reviews’ Reports

| Authors | Appraisal tool | Appraisal rating |
| --- | --- | --- |
| Brea-Gómez et al. (2021) | Downs–Black quality assessment method | Fourteen studies were evaluated, two were classified as excellent (26–28), seven as good (20–25), four as fair (19–15), and one as poor (-14). The mean score of the included studies was 20.79 (range, 13–27). |
| Grassini (2022) | Newcastle–Ottawa Scale (NOS) | Only included studies having scores > 5 on the NOS. Nine studies were evaluated. Three studies scored 6, three scored 7, and three scored 8. |
| Li et al. (2024) | Grading of Recommendations, Assessment, Development, and Evaluations (GRADE) | Pain in the short term and disability in the intermediate term were low.  Kinesiophobia in the short and intermediate term, disability in the short term, and pain in the intermediate term were very low. |
| Ye et al. (2023) | Downs and Black quality assessment | Four studies had an average score of 22 and one was 20. |
|  | Physiotherapy Evidence Database scale | Four studies had a score of 9 and one had a score of 5. |
| Zhang et al. (2024) | GRADE | Pain, disability and kinesiophobia were low. |
