# Supplementary material for: Virtual Reality for Patients With Chronic Musculoskeletal Pain and Disability: An Umbrella Review of Systematic Reviews
Source: Health Sci Rep. 2025 Aug 12;8(8):e71163. doi: 10.1002/hsr2.71163 (PMC12343317; doi:10.1002/hsr2.71163)
Supplement: Supplementary file 4 — S4 File. Excluded Studies. [file HSR2-8-e71163-s005.docx]

**Excluded studies with reasons (n=107)**

Aguilar-Lazcano CA, Rechy-Ramirez EJ, Hu H, Rios-Figueroa HV, Marin-Hernandez A. Interaction modalities used in serious games for upper limb rehabilitation: a systematic review. Games Health J. 2019; 8(5): 313-25. doi: <https://doi.org/10.1089/g4h.2018.0129>. **Reason for exclusion:** **no outcome of interest and** **assessed non-chronic primary musculoskeletal pain.**

Ahern, M. M., Dean, L. V., Stoddard, C. C., Agrawal, A., Kim, K., Cook, C. E., & Narciso Garcia, A. (2020). The effectiveness of virtual reality in patients with spinal pain: a systematic review and meta-analysis. *Pain Pract, 20*(6), 656-675. doi:https://doi.org/10.1111/papr.12885. **Reason for exclusion: assessed non-chronic primary musculoskeletal pain.**

Alhagbani A, Williams A. Home-based exergames for older adults balance and falls risk: a systematic review. Phys Occup Ther Geriatr. 2021; 39(3): 241-57. doi: [https://doi.org/](https://doi.org/10.1016/S0006-8993(02)02471-X)[10.1080/02703181.2020.1867286](https://doi.org/10.1080/02703181.2020.1867286). **Reason for exclusion: no outcome of interest**

Axiaq, A., & Dempster, M. (2024). The use of immersive virtual-reality interventions to improve psychological well-being in adult chronic physical illness. *BMJ Support Palliat Care, 14*(Supplement 2), A4. <https://doi.org/10.1136/spcare-2024-PCC.9>. **Reason for exclusion: inadequate information (abstract)**

Baker, N. A., Polhemus, A. H., Haan Ospina, E., Feller, H., Zenni, M., Deacon, M., . . . Driscoll, M. (2022). The state of science in the use of virtual reality in the treatment of acute and chronic pain: a systematic scoping review. *Clin J Pain, 38*(6), 424-441. <https://doi.org/10.1097/AJP.0000000000001029>. **Reason for exclusion: assessed non-adult, assessed non-chronic primary musculoskeletal pain and postoperative pain.**

Blasco J, Igual-Camacho C, Blasco M, Anton-Anton V, Ortiz-Llueca L, Roig-Casasus S. The efficacy of virtual reality tools for total knee replacement rehabilitation: a systematic review. Physiother Theory Pract. 2021; 37(6): 682-92. doi: <https://doi.org/10.1080/09593985.2019.1641865>. **Reason for exclusion: assessed postoperative** **pain.**

Bordeleau M, Stamenkovic A, Tardif PA, Thomas J. The use of virtual reality in back pain rehabilitation: a systematic review and meta-analysis. J Pain. 2022; 23(2): 175-95. doi: <https://doi.org/10.1016/j.jpain.2021.08.001>. **Reason for exclusion: assessed non-interactive** **virtual reality technologies.**

Byra J, Czernicki K. The effectiveness of virtual reality rehabilitation in patients with knee and hip osteoarthritis. J Clin Med. 2020; 9(8): 2639. doi: <https://doi.org/10.3390/jcm9082639>. **Reason for exclusion: assessed postoperative** **pain.**

Cargnin, Z. A., Schneider, D. G., Souza, M. G. d., Vargas, M. A. d. O., & Tourinho, F. S. V. (2024). Low back pain self-management mobile applications: a systematic review on digital platforms. *Revista da Escola de Enfermagem da USP, 58*, e20230326. <https://doi.org/10.1590/1980-220X-REEUSP-2023-0326en>. **Reason for exclusion: assessed non-interactive virtual reality technologies and no outcome of interest.**

Chaparro-Cárdenas SL, Lozano-Guzmán AA, Ramirez-Bautista JA, Hernández-Zavala A. A review in gait rehabilitation devices and applied control techniques. Disabil Rehabil Assist Technol. 2018; 13(8): 819-34. doi: <https://doi.org/10.1080/17483107.2018.1447611>. **Reason for exclusion: assessed non-chronic primary musculoskeletal pain.**

Charlier N, Zupancic N, Fieuws S, Denhaerynck K, Zaman B, Moons P. Serious games for improving knowledge and self-management in young people with chronic conditions: a systematic review and meta-analysis. J Am Med Inform Assoc. 2016; 23(1): 230-9. doi: <https://doi.org/10.1093/jamia/ocv100>. **Reason for exclusion: no outcome of interest.**

Chen B, Liang RQ, Chen RY, Xu FY. The effect of virtual reality training on the daily participation of patients: a meta-analysis. Complement Ther Med. 2021; 58: 102676. doi: <https://doi.org/10.1016/j.ctim.2021.102676>. **Reason for exclusion: no outcome of interest.**

Chen T, Or CK, Chen J. Effects of technology-supported exercise programs on the knee pain, physical function, and quality of life of individuals with knee osteoarthritis and/or chronic knee pain: a systematic review and meta-analysis of randomized controlled trials. J Am Med Inform Assoc. 2021; 28(2): 414-23. doi: <https://doi.org/10.1093/jamia/ocaa282>. **Reason for exclusion: assessed non-interactive virtual reality technologies.**

Choi, T., Heo, S., Choi, W., & Lee, S. (2023). A systematic review and meta-analysis of the effectiveness of virtual reality-based rehabilitation therapy on reducing the degree of pain experienced by individuals with low back pain. *Int J Environ Res Public Health,* 20(4), 3502. <https://doi.org/10.3390/ijerph20043502>. **Reason for exclusion: Unspecified pain duration.**

Coe-O’Brien R, Joseph L, Kuisma R, Paungmali A, Sitilertpisan P, Pirunsan U. Outcome measures used in smartphone applications for the management of low back pain: a systematic scoping review. Health Inf Sci Syst. 2020; 8(1): 5. doi: <https://doi.org/10.1007/s13755-019-0097-x>. **Reason for exclusion: no outcome of interest.**

Collado-Mateo D, Lavín-Pérez AM, Fuentes García JP, García-Gordillo MÁ, Villafaina S. Effects of equine-assisted therapies or horse-riding simulators on chronic pain: a systematic review and meta-analysis. Medicina (Kaunas). 2020; 56(9): 444. doi: <https://doi.org/10.3390/medicina56090444>. **Reason for exclusion: assessed non-interactive virtual reality technologies and assessed non-chronic primary musculoskeletal pain.**

Cortés-Pérez I, Zagalaz-Anula N, Ibancos-Losada MdR, Nieto-Escámez FA, Obrero-Gaitán E, Osuna-Pérez MC. Virtual reality-based therapy reduces the disabling impact of fibromyalgia syndrome in women: systematic review with meta-analysis of randomized controlled trials. J Pers Med. 2021; 11(11): 1167. doi: <https://doi.org/10.3390/jpm11111167>. **Reason for exclusion: assessed non-chronic primary musculoskeletal pain.**

Cuevas-Lara C, Izquierdo M, de Asteasu MLS, Ramírez-Vélez R, Zambom-Ferraresi F, Zambom-Ferraresi F, et al. Impact of game-based interventions on health-related outcomes in hospitalized older patients: a systematic review. J Am Med Dir Assoc. 2021; 22(2): 364-71. doi: <https://doi.org/10.1016/j.jamda.2020.07.027>. **Reason for exclusion: assessed non-chronic primary musculoskeletal pain.**

Cuevas-Lara C, Sáez de Asteasu ML, Ramírez-Vélez R, Izquierdo M, Zambom-Ferraresi F, Antoñanzas-Valencia C, et al. Effects of game-based interventions on functional capacity in acutely hospitalised older adults: results of an open-label non-randomised clinical trial. Age Ageing. 2022; 51(1): afab247. doi: <https://doi.org/10.1093/ageing/afab247>. **Reason for exclusion: not a systematic review.**

Cugusi L, Prosperini L, Mura G. Exergaming for quality of life in people living with chronic diseases: a systematic review and meta-analysis. PM R. 2021; 13(7): 756-80. doi: <https://doi.org/10.1002/pmrj.12444>. **Reason for exclusion: no outcome of interest and assessed non-chronic primary musculoskeletal pain.**

Dantas LO, Carvalho C, Prando BC, McAlindon TE, da Silva Serrao PRM. Mobile health technologies for the management of rheumatic diseases: a systematic review of online stores in Brazil. Clin Rheumatol. 2021; 40(7): 2601-9. doi: <https://doi.org/10.1007/s10067-020-05561-y>. **Reason for exclusion: assessed non-interactive virtual reality technologies, application languages not in English, and no outcome of interest.**

Dascal J, Reid M, Ishak WW, Spiegel B, Recacho J, Rosen B, et al. Virtual reality and medical inpatients: a systematic review of randomized, controlled trials. Innov Clin Neurosci. 2017; 14(1-2): 14-21. **Reason for exclusion: assessed non-chronic primary musculoskeletal pain.**

Davergne T, Pallot A, Dechartres A, Fautrel B, Gossec L. Use of wearable activity trackers to improve physical activity behavior in patients with rheumatic and musculoskeletal diseases: a systematic review and meta‐analysis. Arthritis Care Res (Hoboken). 2019; 71(6): 758-67. doi: <https://doi.org/10.1002/acr.23752>. **Reason for exclusion: assessed non-interactive virtual reality technologies.**

De La Vega R, Miró J. mHealth: a strategic field without a solid scientific soul. a systematic review of pain-related apps. PLoS One. 2014; 9(7): e101312. doi: <https://doi.org/10.1371/journal.pone.0101312>. **Reason for exclusion: no outcome of interest, assessed non-interactive virtual reality technologies, age range, and assessed non-chronic primary musculoskeletal pain.**

Didyk, C., Lewis, L. K., & Lange, B. (2022). Effectiveness of smartphone apps for the self-management of low back pain in adults: a systematic review. *Disabil Rehabil, 44*(25), 7781-7790. <https://doi.org/10.1080/09638288.2021.2005161>. **Reason for exclusion: assessed non-interactive virtual reality technologies.**

Donegan, T., Ryan, B. E., Sanchez-Vives, M. V., & Swidrak, J. (2022). Altered bodily perceptions in chronic neuropathic pain conditions and implications for treatment using immersive virtual reality. *Front Hum Neurosci, 16*, 1024910. <https://doi.org/10.3389/fnhum.2022.1024910>. **Reason for exclusion: assessed non-chronic primary musculoskeletal pain.**

Dy, M., Olazo, K., Lisker, S., Brown, E., Saha, A., Weinberg, J., & Sarkar, U. (2023). Virtual reality for chronic pain management among historically marginalized populations: systematic review of usability studies. *J Med Internet Res,25*, e40044. <https://doi.org/10.2196/40044>. **Reason for exclusion: assessed non-chronic primary musculoskeletal pain.**

Eccleston C, Fisher E, Craig L, Duggan GB, Rosser BA, Keogh E. Psychological therapies (Internet‐delivered) for the management of chronic pain in adults. Cochrane Database Syst Rev. 2014; 2014(2): CD010152. doi: <https://doi.org/10.1002/14651858.CD010152.pub2>. **Reason for exclusion: assessed non-interactive virtual reality technologies and** **assessed non-chronic primary musculoskeletal pain.**

Elaraby, A., Shahien, M., Jahan, A. M., Etoom, M., & Bekhet, A. H. (2023). The efficacy of virtual reality training in the rehabilitation of orthopedic ankle injuries: a systematic review and meta-analysis. *Adv Rehabil Sci Pract, 12*. <https://doi.org/10.1177/11795727231151636>. **Reason for exclusion: assessed non-chronic primary musculoskeletal pain.**

Faiz D, Nor Rumaizah MN, Puteri Sofia MK, Mohd Shahir A. Utilising virtual reality in pain management: a systematic review. Med Health. 2021; 16(2): 52-71. doi: [[https://doi.org/](https://doi.org/10.1016/S0006-8993(02)02471-X)10.17576/MH.2021.1602.04](https://doi-org.nottingham.idm.oclc.org/10.17576/MH.2021.1602.04). **Reason for exclusion: assessed non-adult and non-chronic primary musculoskeletal pain.**

Fang Q, Ghanouni P, Anderson SE, Touchett H, Shirley R, Fang F, et al. Effects of exergaming on balance of healthy older adults: a systematic review and meta-analysis of randomized controlled trials. Games Health J. 2020; 9(1): 11-23. doi: <https://doi.org/10.1089/g4h.2019.0016>. **Reason for exclusion: assessed non-chronic primary musculoskeletal pain and no outcome of interest.**

Fernandes CS, Magalhães B, Gomes JA, Santos C. Exergames to improve rehabilitation after anterior cruciate ligament injury: systematic review and GRADE evidence synthesis. Int J Orthop Trauma Nurs. 2021; 44: 100917. doi: <https://doi.org/10.1016/j.ijotn.2021.100917>. **Reason for exclusion: assessed postoperative** **pain.**

Fernandes, C. S., Magalhaes, B., Gomes, J. A., & Santos, C. (2022). Exergames to improve rehabilitation for shoulder injury: systematic review and GRADE evidence synthesis. *Rehabil Nurs, 47*(4), 147-159. <https://doi.org/10.1097/RNJ.0000000000000374>. **Reason for exclusion: assessed non-chronic primary musculoskeletal pain.**

Gao, D., Su, Y. Q., Zhang, X., Li, H. S., & Luo, H. C. (2024). The application of virtual reality meditation and mind-body exercises among older adults. *Front Psychol, 15*. <https://doi.org/10.3389/fpsyg.2024.1303880>. **Reason for exclusion: not a systematic review.**

Gava, V., Fialho, H. R. F., Calixtre, L. B., Barbosa, G. M., & Kamonseki, D. H. (2022). Effects of gaming on pain-related fear, pain catastrophizing, anxiety, and depression in patients with chronic musculoskeletal pain: a systematic review and meta-analysis. *Games Health J, 11*(6), 369-384. <https://doi.org/10.1089/g4h.2021.0232>. **Reason for exclusion: assessed non-chronic primary musculoskeletal pain.**

Giravi, H. Y., Biskupiak, Z., Tyler, L. S., & Bulaj, G. (2022). Adjunct digital interventions improve opioid-based pain management: impact of virtual reality and mobile applications on patient-centered pharmacy care. *Fron Digit Health, 4*. <https://doi.org/10.3389/fdgth.2022.884047>. **Reason for exclusion: assessed non-chronic primary musculoskeletal pain.**

Goudman, L., Jansen, J., Billot, M., Vets, N., De Smedt, A., Roulaud, M., . . . Moens, M. (2022). Virtual reality applications in chronic pain management: systematic review and meta-analysis. *JMIR Serious Games, 10*(2). <https://doi.org/10.2196/34402>. **Reason for exclusion: assessed non-chronic primary musculoskeletal pain.**

Grainger R, Townsley H, White B, Langlotz T, Taylor WJ. Apps for people with rheumatoid arthritis to monitor their disease activity: a review of apps for best practice and quality. JMIR Mhealth Uhealth. 2017; 5(2): e7. doi: <https://doi.org/10.2196/mhealth.6956>. **Reason for exclusion: no outcome of interest.**

Guede-Rojas, F., Andrades-Torres, B., Aedo-Diaz, N., Gonzalez-Koppen, C., Munoz-Fuentes, M., Enriquez-Enriquez, D., . . . Fuentes-Contreras, J. (2024). Effects of exergames on rehabilitation outcomes in patients with osteoarthritis. A systematic review. *Disabil Rehabil,* 1-14. <https://doi.org/10.1080/09638288.2024.2368057>. **Reason for exclusion: Unspecified pain duration**

Gumaa M, Khaireldin A, Rehan Youssef A. Validity and reliability of interactive virtual reality in assessing the musculoskeletal system: a systematic review. Curr Rev Musculoskelet Med. 2021; 14(2): 130-44. doi: <https://doi.org/10.1007/s12178-021-09696-6>. **Reason for exclusion: no outcome of interest.**

Gumaa M, Rehan Youssef A. Is virtual reality effective in orthopedic rehabilitation? Systematic review and meta-analysis. Phys Ther. 2019; 99(10): 1304-25. doi: <https://doi.org/10.1093/ptj/pzz093>. **Reason for exclusion: assessed postoperative pain.**

Guo, L., Li, S., Xie, S., Bian, L., & Shaharudin, S. (2024). The impact of digital healthcare systems on pain and body function in patients with knee joint pain: a systematic review and meta-analysis. *Sci Rep, 14*(1), 3310. <https://doi.org/10.1038/s41598-024-53853-z>. **Reason for exclusion: assessed non-interactive virtual reality technologies.**

Guo, Q., Zhang, L., Gui, C., Chen, G., Chen, Y., Tan, H., . . . Gao, Q. (2023). Virtual reality intervention for patients with neck pain: systematic review and meta-analysis of randomized controlled trials. *J Med Internet Res*, 25, e38256. <https://doi.org/10.2196/38256>. **Reason for exclusion: Unspecified pain duration**

Hong, S. J., Park, J., Park, S., Eze, B., Dorsey, S. G., Starkweather, A., & Kim, K. (2024). Software-based interventions for low back pain management: a systematic review and meta-analysis. *J Nurs Scholarsh, 56*(2), 206-226. <https://doi.org/10.1111/jnu.12937>. **Reason for exclusion: assessed non-interactive virtual reality technologies.**

Huang, Q., Lin, J., Han, R., Peng, C., & Huang, A. (2022). Using virtual reality exposure therapy in pain management: a systematic review and meta-analysis of randomized controlled trials. *Value Health, 25*(2), 288-301. <https://doi.org/10.1016/j.jval.2021.04.1285>. **Reason for exclusion: assessed non-chronic primary musculoskeletal pain.**

Hussain, A., Haroon, H., Ahmed, A., & Gilani, S. A. (2022). Digital technologies in management of chronic pain - a systematic review. *J Pak Med Assoc, 72*(6), 1158-1165. <https://doi.org/10.47391/JPMA.3885>. **Reason for exclusion: unable to access the full-text.**

Iversen MD, Connors ME, Menapace MR, Samson AJ, Tessitore E. Technology applications to improve health outcomes and self-management in patients with arthritis. Int J Clin Rheumatol. 2014; 9(5): 487. doi: <https://doi.org/10.2217/IJR.14.35>. **Reason for exclusion: assessed non-interactive virtual reality technologies.**

Jang, S., Lee, B., Lee, E. J., Kim, J., Lee, J. I., Lim, J. Y., . . . Jang, S. (2023). A systematic review and meta-analysis of the effects of rehabilitation using digital healthcare on musculoskeletal pain and quality of life. *J Pain Res, 16*, 1877-1894. <https://doi.org/10.2147/JPR.S388757>. **Reason for exclusion: assessed postoperative pain and non-interactive virtual reality technologies.**

Juras G, Brachman A, Michalska J, Kamieniarz A, Pawłowski M, Hadamus A, et al. Standards of virtual reality application in balance training programs in clinical practice: a systematic review. Games Health J. 2019; 8(2): 101-11. doi: <https://doi.org/10.1089/g4h.2018.0034>. **Reason for exclusion: assessed non-chronic primary musculoskeletal pain.**

Kantha, P., Lin, J.-J., & Hsu, W.-L. (2023). The effects of interactive virtual reality in patients with chronic musculoskeletal disorders: a systematic review and meta-analysis. *Games Health J, 12*(1), 1-12. <https://doi.org/10.1089/g4h.2022.0088>. **Reason for exclusion: assessed non-chronic primary musculoskeletal pain.**

Keefe FJ, Huling DA, Coggins MJ, Keefe DF, Rosenthal MZ, Herr NR, et al. Virtual reality for persistent pain: a new direction for behavioral pain management. Pain. 2012; 153(11): 2163-66. doi: <https://doi.org/10.1016/j.pain.2012.05.030>. **Reason for exclusion: not a systematic review.**

Kılıç A, Brown A, Aras I, Hui R, Hare J, Hughes LD, et al. Using virtual technology for fear of medical procedures: a systematic review of the effectiveness of virtual reality-based interventions. Ann Behav Med. 2021; 55(11): 1062-79. doi: <https://doi.org/10.1093/abm/kaab016>. **Reason for exclusion: no outcome of interest.**

Kim, J., Jun, K., Park, S., & Lee, S. W. (2023). Bibliometric analysis of research articles on virtual reality in the field of pain medicine published from 1993 to 2022. *J Pain Res, 16*, 3881-3893. <https://doi.org/10.2147/JPR.S432113>. **Reason for exclusion: assessed non-chronic primary musculoskeletal pain.**

Koumpouros, Y., & Georgoulas, A. (2023). Pain management mobile applications: a systematic review of commercial and research efforts. *Sensors (Basel, Switzerland), 23*(15). <https://doi.org/10.3390/s23156965>. **Reason for exclusion: assessed non-chronic primary musculoskeletal pain and non-interactive** **virtual reality technologies.**

Lee DC, Ham YW, Sung PS. Effect of visual input on normalized standing stability in subjects with recurrent low back pain. Gait Posture. 2012; 36(3): 580-5. doi: <https://doi.org/10.1016/j.gaitpost.2012.05.020>. **Reason for exclusion: not a systematic review.**

Lier, E. J., De Vries, M., Steggink, E. M., Ten Broek, R. P. G., & Van Goor, H. (2023). Effect modifiers of virtual reality in pain management: a systematic review and meta-regression analysis. *Pain, 164*(8), 1658-1665. <https://doi.org/10.1097/j.pain.0000000000002883>. **Reason for exclusion: assessed non-chronic primary musculoskeletal pain.**

Lisi, M. P., Fusaro, M., & Aglioti, S. M. (2024). Visual perspective and body ownership modulate vicarious pain and touch: a systematic review. *Psychon Bull Rev*. <https://doi.org/10.3758/s13423-024-02477-5>. **Reason for exclusion: no outcome of interest.**

Liu, H., Xing, Y., & Wu, Y. (2022). Effect of Wii Fit exercise with balance and lower limb muscle strength in older adults: a meta-analysis. *Front Med, 9*, 812570. <https://doi.org/10.3389/fmed.2022.812570>. **Reason for exclusion: no outcome of interest.**

Luc, A., Tamer, S., Hage, R., Detrembleur, C., & Pitance, L. (2022). Do the kinematics and sensorimotor control of people with chronic non-specific neck pain differ from those of healthy individuals when assessed in an immersive virtual reality environment? A systematic review. *Phys Ther Rev, 27*(6), 430-443. <https://doi.org/10.1080/10833196.2022.2143211>. **Reason for exclusion: no outcome of interest.**

Luo D, Wang P, Lu F, Elias J, Sparks JA, Lee YC. Mobile apps for individuals with rheumatoid arthritis: a systematic review. J Clin Rheumatol. 2019; 25(3): 133-41. doi: <https://doi.org/10.1097/RHU.0000000000000800>. **Reason for exclusion: no outcome of interest.**

Machado GC, Pinheiro MB, Lee H, Ahmed OH, Hendrick P, Williams C, et al. Smartphone apps for the self-management of low back pain: a systematic review. Best Pract Res Clin Rheumatol. 2016; 30(6): 1098-109. doi: <https://doi.org/10.1016/j.berh.2017.04.002>. **Reason for exclusion: no outcome of interest and assessed non-interactive virtual reality technologies.**

Majeed-Ariss R, Baildam E, Campbell M, Chieng A, Fallon D, Hall A, et al. Apps and adolescents: a systematic review of adolescents’ use of mobile phone and tablet apps that support personal management of their chronic or long-term physical conditions. J Med Internet Res. 2015; 17(12): e287. doi: <https://doi.org/10.2196/jmir.5043>. **Reason for exclusion: assessed non-chronic primary musculoskeletal pain, adolescents, and non-interactive virtual reality technologies.**

Mallari B, Spaeth EK, Goh H, Boyd BS. Virtual reality as an analgesic for acute and chronic pain in adults: a systematic review and meta-analysis. J Pain Res. 2019; 12: 2053-85. doi: [https://doi.org/](https://doi.org/10.1016/S0006-8993(02)02471-X) 10.2147/JPR.S200498. **Reason for exclusion: assessed non-chronic primary musculoskeletal pain.**

Martin CL, Bakker CJ, Breth MS, Gao G, Lee K, Lee MA, et al. The efficacy of mobile health interventions used to manage acute or chronic pain: a systematic review. Res Nurs Health. 2021; 44(1): 111-28. doi: <https://doi.org/10.1002/nur.22097>. **Reason for exclusion:** **assessed non-chronic primary musculoskeletal pain and non-interactive virtual reality technologies.**

Mather III RC, Smith JM, Peterson ED. Effects of virtual exercise rehabilitation in-home therapy compared with traditional care after total knee arthroplasty. J Bone Joint Surg Am. 2020; 102(2): 101-9. doi: <https://doi.org/10.2106/JBJS.19.00695>. **Reason for exclusion: not a systematic review and assessed postoperative** **pain.**

Matthie, N. S., Giordano, N. A., Jenerette, C. M., Magwood, G. S., Leslie, S. L., Northey, E. E., . . . Sil, S. (2022). Use and efficacy of virtual, augmented, or mixed reality technology for chronic pain: a systematic review. *Pain Manag, 12*(7), 859-878. <https://doi.org/10.2217/pmt-2022-0030>. **Reason for exclusion: assessed non-chronic primary musculoskeletal pain.**

Mattison, G., Canfell, O., Forrester, D., Dobbins, C., Smith, D., Toyras, J., & Sullivan, C. (2022). The influence of wearables on health care outcomes in chronic disease: systematic review. *J Med Internet Res, 24*(7), e36690. <https://doi.org/10.2196/36690>. **Reason for exclusion: assessed non-chronic primary musculoskeletal pain.**

Meijer HA, Graafland M, Goslings JC, Schijven MP. Systematic review on the effects of serious games and wearable technology used in rehabilitation of patients with traumatic bone and soft tissue injuries. Arch Phys Med Rehabil. 2018; 99(9): 1890-9. doi: <https://doi.org/10.1016/j.apmr.2017.10.018>. **Reason for exclusion: assessed non-adult.**

Mo, N., Feng, J. Y., Liu, H. X., Chen, X. Y., Zhang, H., & Zeng, H. (2023). Effects of exergaming on musculoskeletal pain in older adults: systematic review and meta-analysis. *JMIR Serious Games, 11*. <https://doi.org/10.2196/42944>. **Reason for exclusion: assessed postoperative pain.**

Mollard E, Michaud K. Self-management of rheumatoid arthritis: mobile applications. Curr Rheumatol Rep. 2021; 23(1): 2. doi: <https://doi.org/10.1007/s11926-020-00968-7>. **Reason for exclusion: assessed non-interactive virtual reality technologies.**

Moreau, S., Therond, A., Cerda, I. H., Studer, K., Pan, A., Tharpe, J., . . . Robinson, C. L. (2024). Virtual reality in acute and chronic pain medicine: an updated review. *Curr Pain Headache Rep*. <https://doi.org/10.1007/s11916-024-01246-2>. **Reason for exclusion: not a systematic review.**

Nájera, N. H., Hernández, N. C., & Legleu, C. E. C. (2022). Comparison of tests to measure muscle fatigue in training of male CrossFit athlete: a systematic review. *Retos-Nuevas Tendencias En Educacion Fisica Deporte Y Recreacion*(43), 923-930. **Reason for exclusion: unable to access the English full-text.**

Najm A, Gossec L, Weill C, Benoist D, Berenbaum F, Nikiphorou E. Mobile health apps for self-management of rheumatic and musculoskeletal diseases: a systematic literature review. JMIR Mhealth and Uhealth. 2019; 7(11): e14730. doi: <https://doi.org/10.2196/14730>. **Reason for exclusion: no outcome of interest and assessed non-interactive virtual reality technologies.**

Nussbaum R, Kelly C, Quinby E, Mac A, Parmanto B, Dicianno BE. Systematic review of mobile health applications in rehabilitation. Arch Phys Med Rehabil. 2019; 100(1): 115-27. doi: <https://doi.org/10.1016/j.apmr.2018.07.439>. **Reason for exclusion: no outcome of interest and assessed non-interactive virtual reality technologies.**

Opara, M., & Kozinc, Z. (2024). Virtual reality training for management of chronic neck pain: a systematic review with meta-analysis. *Eur J Physiother, 26*(3), 135-147. <https://doi.org/10.1080/21679169.2023.2215831>. **Reason for exclusion: assessed non-chronic primary musculoskeletal pain.**

Peng L, Zeng Y, Wu Y, Si H, Shen B. Virtual reality-based rehabilitation in patients following total knee arthroplasty: a systematic review and meta-analysis of randomized controlled trials. Chin Med J (Engl). 2022; 135(2): 153-63. doi: <https://doi.org/10.1097/CM9.0000000000001847>. **Reason for exclusion:** **assessed postoperative** **pain.**

Pereira MF, Prahm C, Kolbenschlag J, Oliveira E, Rodrigues NF. Application of AR and VR in hand rehabilitation: a systematic review. J Biomed Inform. 2020; 111: 103584. doi: <https://doi.org/10.1016/j.jbi.2020.103584>. **Reason for exclusion: assessed non-chronic primary musculoskeletal pain.**

Pfeifer AC, Uddin R, Schröder-Pfeifer P, Holl F, Swoboda W, Schiltenwolf M. Mobile application-based interventions for chronic pain patients: a systematic review and meta-analysis of effectiveness. J Clin Med. 2020; 9(11): 3557. doi: <https://doi.org/10.3390/jcm9113557>. **Reason for exclusion: assessed non-chronic primary musculoskeletal pain, non-interactive virtual reality technologies, and age range 6–80 years.**

Phuphanich ME, Sinha KR, Truong M, Pham QG. Telemedicine for musculoskeletal rehabilitation and orthopaedic postoperative rehabilitation. Phys Med Rehabil Clin N Am. 2021;32(2): 319-53. doi: <https://doi.org/10.1016/j.pmr.2020.12.004>. **Reason for exclusion: not a systematic review and assessed postoperative** **pain.**

Portelli P, Eldred C. A quality review of smartphone applications for the management of pain. Br J Pain. 2016; 10(3): 135-40. doi: <https://doi.org/10.1177/2049463716638700>. **Reason for exclusion: assessed non-interactive virtual reality technologies.**

Qian J, McDonough DJ, Gao Z. The effectiveness of virtual reality exercise on individual’s physiological, psychological and rehabilitative outcomes: a systematic review. Int J Environ Res Public Health. 2020; 17(11): 4133. doi: <https://doi.org/10.3390/ijerph17114133>. **Reason for exclusion: assessed non-chronic primary musculoskeletal pain and no outcome of interest.**

Ren C, Liu T, Zhang J. Horse‐riding simulators in treatment of chronic low back pain: a meta‐analysis. Int J Clin Pract. 2021; 75(7): e14198. doi: <https://doi.org/10.1111/ijcp.14198>. **Reason for exclusion: inadequate information regarding the risk of bias results.**

Ridout B, Kelson J, Campbell A, Steinbeck K. Effectiveness of virtual reality interventions for adolescent patients in hospital settings: systematic review. J Med Internet Res. 2021; 23(6): e24967. doi: <https://doi.org/10.2196/24967>. **Reason for exclusion:** **assessed non-chronic primary musculoskeletal pain and adolescents.**

Rigby BR, Grandjean PW. The efficacy of equine-assisted activities and therapies on improving physical function. J Altern Complement Med. 2016; 22(1): 9-24. doi: <https://doi.org/10.1089/acm.2015.0171>. **Reason for exclusion: assessed non-chronic primary musculoskeletal pain.**

Ritu, S., Nithya, M. S., & Ranjana. (2023). Examining the benefits and effectiveness of using horses for therapeutic and recreational activities with aged patients. *REDVET, 24*(3), 35-45. Retrieved from <https://www.veterinaria.org/index.php/REDVET/article/view/393>. **Reason for exclusion: assessed non-interactive virtual reality technologies.**

Safari R, Jackson J, Sheffield D. Digital self-management interventions for people with osteoarthritis: a systematic review with meta-analysis. J Med Internet Res. 2020; 22(7): e15365. doi: <https://doi.org/10.2196/15365>. **Reason for exclusion: assessed non-interactive virtual reality technologies.**

Saragih, I. D., Suarilah, I., Saragih, I. S., Lin, Y.-K., & Lin, C.-J. (2024). Efficacy of serious games for chronic pain management in older adults: a systematic review and meta-analysis. *J Clin Nurs, 33*(3), 1185-1194. <https://doi.org/10.1111/jocn.17012>. **Reason for exclusion: assessed postoperative pain**

Schroder, D., Wrona, K. J., Muller, F., Heinemann, S., Fischer, F., & Dockweiler, C. (2023). Impact of virtual reality applications in the treatment of anxiety disorders: a systematic review and meta-analysis of randomized-controlled trials. *J Behav Ther Exp Psychiatry 81*, 101893. <https://doi.org/10.1016/j.jbtep.2023.101893>. **Reason for exclusion: assessed non-chronic primary musculoskeletal pain and no outcome of interest**

Schuermans, J., Van Hootegem, A., Van den Bossche, M., Van Gendt, M., Witvrouw, E., & Wezenbeek, E. (2022). Extended reality in musculoskeletal rehabilitation and injury prevention - a systematic review. *Phys Ther Sport, 55*, 229-240. **Reason for exclusion:** **assessed** **non-adult.**

Segura‐Ortí E, García‐Testal A. Intradialytic virtual reality exercise: increasing physical activity through technology. Semin Dial. 2019; 32(4): 331-5. doi: <https://doi.org/10.1111/sdi.12788>. **Reason for exclusion: assessed non-chronic primary musculoskeletal pain.**

Shetty, A., Delanerolle, G., Zeng, Y., Shi, J. Q., Ebrahim, R., Pang, J., . . . Phiri, P. (2022). A systematic review and meta-analysis of digital application use in clinical research in pain medicine. *Front Digit Health, 4*, 850601. <https://doi.org/10.3389/fdgth.2022.850601>. **Reason for exclusion: assessed non-chronic primary musculoskeletal pain and non-interactive virtual reality technologies.**

Staiano AE, Flynn R. Therapeutic uses of active videogames: a systematic review. Games Health J. 2014; 3(6): 351-65. doi: <https://doi.org/10.1089/g4h.2013.0100>. **Reason for exclusion: assessed non-chronic primary musculoskeletal pain.**

Stark, C., Cunningham, J., Turner, P., Johnson, M. A., & Backer, H. C. (2022). App-based rehabilitation in back pain, a systematic review. *J Pers Med, 12*(10), 1558. <https://doi.org/10.3390/jpm12101558>. **Reason for exclusion: assessed non-interactive virtual reality technologies and postoperative pain.**

Subnis UB, Starkweather A, Menzies V. A current review of distraction-based interventions for chronic pain management. Eur J Integrat Med. 2016; 8(5): 715-22. doi: <https://doi.org/10.1016/j.eujim.2016.08.162>. **Reason for exclusion: assessed non-chronic primary musculoskeletal pain.**

Tabacof L, Baker TS, Durbin JR, Desai V, Zeng Q, Sahasrabudhe A, et al. Telehealth treatment for nonspecific low back pain: a review of the current state of mobile health. PM R. 2021; 14(9): 1086-98. doi: <https://doi.org/10.1002/pmrj.12738>. **Reason for exclusion: assessed non-interactive virtual reality technologies.**

Thompson, D., Rattu, S., Tower, J., Egerton, T., Francis, J., & Merolli, M. (2023). Mobile app use to support therapeutic exercise for musculoskeletal pain conditions may help improve pain intensity and self-reported physical function: a systematic review. *J Physiother, 69*(1), 23-34. <https://doi.org/10.1016/j.jphys.2022.11.012>. **Reason for exclusion: assessed non-interactive virtual reality technologies.**

Verbrugghe J, Knippenberg E, Palmaers S, Matheve T, Smeets W, Feys P, et al. Motion detection supported exercise therapy in musculoskeletal disorders: a systematic review. Eur J Phys Rehabil Med. 2018; 54(4): 591-604. doi: <https://doi.org/10.23736/S1973-9087.18.04614-2>. **Reason for exclusion: assessed postoperative pain**

Viana RB, de Oliveira VN, Dankel SJ, Loenneke JP, Abe T, da Silva WF, et al. The effects of exergames on muscle strength: a systematic review and meta‐analysis. Scand J Med Sci Sports. 2021; 31(8): 1592-611. doi: <https://doi.org/10.1111/sms.13964>. **Reason for exclusion: no outcome of interest.**

Vibhuti, Kumar, N., & Kataria, C. (2023). Efficacy assessment of virtual reality therapy for neuromotor rehabilitation in home environment: a systematic review. *Disabil Rehabil Assist Technol, 18*(7), 1200-1220. <https://doi.org/10.1080/17483107.2021.1998674>. **Reason for exclusion: assessed non-chronic primary musculoskeletal pain.**

Wang X, Hunter DJ, Vesentini G, Pozzobon D, Ferreira ML. Technology-assisted rehabilitation following total knee or hip replacement for people with osteoarthritis: a systematic review and meta-analysis. BMC Musculoskelet Disord. 2019; 20(1): 1-17. doi: <https://doi.org/10.1186/s12891-019-2900-x>. **Reason for exclusion: assessed postoperative** **pain.**

Wang, S., Sun, J., Yin, X., & Li, H. (2023). Effect of virtual reality technology as intervention for people with kinesiophobia: a meta‐analysis of randomised controlled trials. *J Clin Nurs, 32*(13/14), 3074-3086. <https://doi.org/10.1111/jocn.16397>. **Reason for exclusion: assessed non-chronic primary musculoskeletal pain.**

Wen, Y., Shen, X., & Shen, Y. (2024). Improving immersive experiences in virtual natural setting for public health and environmental design: a systematic review and meta-analysis of randomized controlled trials. *PLoS One, 19*(4 April), e0297986. <https://doi.org/10.1371/journal.pone.0297986>. **Reason for exclusion: assessed non-chronic primary musculoskeletal pain.**

Wittkopf PG, Lloyd DM, Coe O, Yacoobali S, Billington J. The effect of interactive virtual reality on pain perception: a systematic review of clinical studies. Disabil Rehabil. 2020; 42(26): 3722-33. doi: <https://doi.org/10.1080/09638288.2019.1610803>. **Reason for exclusion: assessed non-chronic primary musculoskeletal pain.**

Wong, K. P., Tse, M. M. Y., & Qin, J. (2022). Effectiveness of virtual reality-based interventions for managing chronic pain on pain reduction, anxiety, depression and mood: a systematic review. *Healthcare, 10*(10). <https://doi.org/10.3390/healthcare10102047>. **Reason for exclusion: assessed non-chronic primary musculoskeletal pain.**

Xia, W., Ding, J., Yan, Y., Chen, F., Yan, M., & Xu, X. (2024). Effectiveness of virtual reality technology in symptom management of patients at the end of life: a systematic review and meta-analysis. *J AM Med Dir Assoc*, 105086. <https://doi.org/10.1016/j.jamda.2024.105086>. **Reason for exclusion: assessed non-chronic primary musculoskeletal pain.**

Zhao P, Yoo I, Lancey R, Varghese E. Mobile applications for pain management: an app analysis for clinical usage. BMC Med Inform Decis Mak. 2019; 19(1): 106. doi: <https://doi.org/10.1186/s12911-019-0827-7>. **Reason for exclusion: assessed non-chronic primary musculoskeletal pain and non-interactive virtual reality technologies.**

Zhu, D., Zhao, J., Wang, M., Cao, B., Zhang, W., Li, Y., . . . Han, T. (2024). Rehabilitation applications based on behavioral therapy for people with knee osteoarthritis: systematic review. *JMIR Mhealth Uhealth, 12*, e53798. <https://doi.org/10.2196/53798>. **Reason for exclusion: assessed non-interactive virtual reality technologies.**
