# Supplementary material for: Virtual Reality for Patients With Chronic Musculoskeletal Pain and Disability: An Umbrella Review of Systematic Reviews
Source: Health Sci Rep. 2025 Aug 12;8(8):e71163. doi: 10.1002/hsr2.71163 (PMC12343317; doi:10.1002/hsr2.71163)
Supplement: Supplementary file 5 — S5 File. GRADE Results. [file HSR2-8-e71163-s002.docx]

Table 1: Certainty of evidence using the Grading of Recommendations, Assessment, Development, and Evaluations (GRADE)

| Condition ‘comparisons’ (reported review) | Outcome ‘measure, score’ | Trials (participants) | Effect estimates 95% CI, p-value | Certainty of evidence | Comments |
| --- | --- | --- | --- | --- | --- |
| Chronic neck pain (CNP): ‘virtual reality (VR) vs control’ (Grassini, 2022) | Pain: ‘visual analogue scale (VAS), 0–100’ | 3 (146) | (mean difference [MD] = −8.80, 95% confidence interval [CI] = −12.49 to −3.67, p < 0.05), decreased VAS score in favour of VR group (VRG). | Low | Risk of bias: 3/3 at high risk of bias (downgraded)  Inconsistency: I^2^=0% (not downgraded)  Indirectness: N/A  Imprecision: n < 400 (downgraded)  Publication Bias: N/A |
| CNP: ‘VR pre-intervention vs post-intervention’ (Grassini, 2022) | Pain: ‘VAS, 0–100’ | 3 (141) | (MD = 26.24, 95% CI = 13.34 to 39.13, p < 0.05), decreased VAS score in favour of post-intervention. | Very low | Risk of bias: 3/3 at high risk of bias (downgraded)  Inconsistency: I^2^=82% (downgraded)  Indirectness: N/A  Imprecision: n < 400 (downgraded)  Publication Bias: N/A |
| CNP: ‘VR vs. control group’ at short term (Hao, He, Chen, & Remis, 2024) | Pain: ‘VAS, 0–10’ | 6 (275) | (MD = −0.94, 95% CI = −1.31 to −0.58, p < 0.01), in favour of VRG. | Low | Risk of bias: 6/6 at high risk of bias (downgraded)  Inconsistency: I^2^=0% (not downgraded)  Indirectness: N/A  Imprecision: n < 400 (downgraded)  Publication Bias: N/A |
| CNP: ‘VR vs. control group’ at intermediate term (Hao et al., 2024) | Pain: ‘VAS, 0–10’ | 4 (122) | (MD = −0.61, 95% CI = −0.27 to 0.05, p = 0.07), no significant difference | Low | Risk of bias: 4/4 at high risk of bias (downgraded)  Inconsistency: I^2^=41.4% (not downgraded)  Indirectness: N/A  Imprecision: n < 400 (downgraded)  Publication Bias: N/A |
| CNP: ‘VR pre-intervention vs post-intervention’ (Ye, Koh, Jaiswal, Soomal, & Kumbhare, 2023) | Pain: ‘VAS, not reported (NR)’ | 4 (154) | (MD = 2.13, 95% CI = 1.11 to 3.15, p ˂ 0.0001), in favour of post-VR intervention. | Low | Risk of bias: 3/3 at low risk of bias (not downgraded)  Inconsistency: I^2^=84% (downgraded)  Indirectness: N/A  Imprecision: n < 400 (downgraded)  Publication Bias: N/A |
| CNP: ‘VR vs. control group’ (Ye et al., 2023) | Pain: ‘VAS, NR’ | 4 (155) | (MD = 0.58, 95% CI = 0.25 to 0.91, p = 0.0005), in favour of VRG. | Moderate | Risk of bias: 4/4 at low risk of bias (not downgraded)  Inconsistency: I^2^=2% (not downgraded)  Indirectness: N/A  Imprecision: n ˂ 400 (downgraded)  Publication Bias: N/A |
| CNP: ‘VR vs. control group’ (Zhang et al., 2024) | Pain: ‘VAS, 0–10’ | 4 (180) | (Weighted MD [WMD] = −1.22, 95% CI = −1.80 to −0.63, p ˂ 0.00001), in favour of VRG. | Low | Risk of bias: 4/4 at high risk of bias (downgraded)  Inconsistency: I^2^=0% (not downgraded)  Indirectness: N/A  Imprecision: n < 400 (downgraded)  Publication Bias: N/A |
| CNP: ‘VR pre-intervention vs post-intervention’ (Ye et al., 2023) | Disability ‘NDI, NR’ | 3 (120) | (MD = 2.08, 95% CI = 0.40 to 3.67, p = 0.02), in favour of post-VR intervention. | Low | Risk of bias: 3/3 at low risk of bias (not downgraded)  Inconsistency: I^2^=92% (downgraded)  Indirectness: N/A  Imprecision: n < 400 (downgraded)  Publication Bias: N/A |
| CNP: ‘VR vs. control group’ (Ye et al., 2023) | Disability ‘NDI, NR’ | 3 (121) | (MD = 0.54, 95% CI = −0.15 to 1.24, p = 0.13), no significant difference. | Low | Risk of bias: 3/3 at low risk of bias (not downgraded)  Inconsistency: I^2^=72% (downgraded)  Indirectness: N/A  Imprecision: n < 400 (downgraded)  Publication Bias: N/A |
| CNP: ‘VR vs. control' (Grassini, 2022) | Disability: ‘Neck Disability Index (NDI), 0–50’ | 3 (136) | (MD = −2.87, 95% CI = −4.36 to −1.39, p = 0.0002), in favour of VRG. | Low | Risk of bias: 3/3 at high risk of bias (downgraded)  Inconsistency: I^2^=33% (not downgraded)  Indirectness: N/A  Imprecision: n < 400 (downgraded)  Publication Bias: N/A |
| CNP: ‘VR vs. control group’ at short term (Hao et al., 2024) | Disability ‘NDI, 0-50’ | 5 (241) | (MD = −2.16, 95% CI = −3.50 to −0.82, p < 0.01), in favour of VRG. | Low | Risk of bias: 5/5 at high risk of bias (downgraded)  Inconsistency: I^2^=15.4% (not downgraded)  Indirectness: N/A  Imprecision: n < 400 (downgraded)  Publication Bias: N/A |
| CNP: ‘VR vs. control group’ at intermediate term (Hao et al., 2024) | Disability ‘NDI, 0-50’ | 4 (204) | (MD = −2.95, 95% CI = −4.93 to −0.97, p < 0.01), in favour of VRG. | Low | Risk of bias: 4/4 at high risk of bias (downgraded)  Inconsistency: I^2^=36.2% (not downgraded)  Indirectness: N/A  Imprecision: n < 400 (downgraded)  Publication Bias: N/A |
| CNP: VR vs. control (Grassini, 2022) | Kinesiophobia: ‘Tampa Scale for Kinesiophobia (TSK), 0–68’ | 2 (94) | (MD = −0.28, 95% CI = −3.46 to 2.90, p = 0.85), no significant difference | Low | Risk of bias: 2/2 at high risk of bias (downgraded)  Inconsistency: I^2^=0% (not downgraded)  Indirectness: N/A  Imprecision: n < 400 (downgraded)  Publication Bias: N/A |
| CNP: ‘VR vs. control group’ at short term (Hao et al., 2024) | Kinesiophobia ‘TSK, NR’ | 3 (166) | (MD = −1.04, 95% CI = −3.54 to 1.45, p = 0.41), no significant difference. | Low | Risk of bias: 3/3 at high risk of bias (downgraded)  Inconsistency: I^2^=0% (not downgraded)  Indirectness: N/A  Imprecision: n < 400 (downgraded)  Publication Bias: N/A |
| CNP: ‘VR vs. control group’ at intermediate term (Hao et al., 2024) | Kinesiophobia ‘TSK, NR’ | 3 (162) | (MD = −2.10, 95% CI = −5.46 to 1.25, p = 0.22), no significant difference. | Very low | Risk of bias: 3/3 at high risk of bias (downgraded)  Inconsistency: I^2^=51.5% (downgraded)  Indirectness: N/A  Imprecision: n < 400 (downgraded)  Publication Bias: N/A |
| Chronic low back pain (CLBP): VR vs. exercise intervention (Grassini, 2022) | Pain: ‘VAS, 0–100’ | 2 (62) | (MD = 1.92, 95% CI = −10.52 to 14.35, p = 0.76), no significant difference. | Very low | Risk of bias: 1/2 at high risk of bias (downgraded)  Inconsistency: I^2^=74% (downgraded)  Indirectness: N/A  Imprecision: n < 400 (downgraded)  Publication Bias: N/A |
| CLBP: VR vs. control (Grassini, 2022) | Pain: ‘VAS, 0–100’ | 4 (158) | (MD = −10.15, 95% CI = −23.42 to 3.12, p = 0.13), no significant difference | Very low | Risk of bias: 2/4 at high risk of bias (downgraded)  Inconsistency: I^2^=95% (downgraded)  Indirectness: N/A  Imprecision: n < 400 (downgraded)  Publication Bias: N/A |
| CLBP: ‘VR pre-intervention vs post-intervention’ (Grassini, 2022) | Pain: ‘VAS, 0–100’ | 3 (106) | (MD = 32.96, 95% CI = 10.34 to 55.57, p < 0.05), decreased VAS score in favour of post-intervention. | Very low | Risk of bias: 2/3 at high risk of bias (downgraded)  Inconsistency: I^2^=97% (downgraded)  Indirectness: N/A  Imprecision: n < 400 (downgraded)  Publication Bias: N/A |
| CLBP: ‘VR vs. control group’ (Zhang et al., 2024) | Pain: ‘VAS, 0–10’ | 11 (596) | (WMD = −1.74, 95% CI = −2.28 to −1.20, p ˂ 0.00001), in favour of VRG. | Low | Risk of bias: 6/11 at high risk of bias (downgraded)  Inconsistency: I^2^=92% (downgraded)  Indirectness: N/A  Imprecision: n ˃ 400 (not downgraded)  Publication Bias: NR (not downgraded) |
| CLBP: ‘VR vs. control group’ (Kumar, Vatkar, Kataria, Dhatt, & Baburaj, 2024) | Pain: ‘NR, NR’ | 7 (507) | (MD = 1.99, 95% CI = 0.60 to 3.38, p < 0.005), in favour of VRG. | Low | Risk of bias: 2/7 at high risk of bias (downgraded)  Inconsistency: I^2^=95% (downgraded)  Indirectness: N/A  Imprecision: n ˃ 400 (not downgraded)  Publication Bias: N/A |
| CLBP: ‘VR vs. control group’ at short term (Li et al., 2024) | Pain: ‘VAS, 11-point Numerical Pain Rating Scale (11-NPRS) and Defense and Veterans Pain Rating Scale (DVPRS), 0–10’ | 19 (879) | (MD = −1.43, 95% CI = −1.86 to −1.00, p ˂ 0.001), in favour of VRG. | Low | Risk of bias: 18/19 at some concerns risk of bias (downgraded)  Inconsistency: I^2^=95% (downgraded)  Indirectness: N/A  Imprecision: n > 400 (not downgraded)  Publication Bias: NR |
| CLBP: ‘VR vs. control group’ at intermediate term (Li et al., 2024) | Pain: ‘VAS, 11-NPRS and DVPRS, 0–10’ | 7 (393) | (MD = −0.57, 95% CI = −1.36 to 0.22, p = 0.16), no significant difference. | Very low | Risk of bias: 7/7 at some concerns risk of bias (downgraded)  Inconsistency: I^2^=99% (downgraded)  Indirectness: N/A  Imprecision: n < 400 (downgraded)  Publication Bias: N/A |
| CLBP: ‘VR vs. control’ (Brea-Gómez et al., 2021) | Pain: ‘VAS, 0–10’, four weeks intervention duration. | 4 (231) | (standardised MD [SMD] = −3.38, 95% CI = −5.06 to −1.70, p < 0.0001), in favour of VRG. | Low | Risk of bias: 4/4 at low risk of bias (not downgraded)  Inconsistency: I^2^=94% (downgraded)  Indirectness: N/A  Imprecision: n < 400 (downgraded)  Publication Bias: N/A |
| CLBP: ‘VR-based Prokin System vs. control’ (Brea-Gómez et al., 2021) | Pain: ‘VAS, 0–10’ | 3 (212) | (SMD = −3.96, 95% CI = −5.71 to –2.21, p < 0.00001), in favour of VRG | Low | Risk of bias: 3/3 at low risk of bias (not downgraded)  Inconsistency: I^2^=93% (downgraded)  Indirectness: N/A  Imprecision: n < 400 (downgraded)  Publication Bias: N/A |
| CLBP: ‘VR-based Prokin System vs. control for a 4-week intervention’ at intermediate term (Brea-Gómez et al., 2021) | Pain: ‘VAS, 0–10’ | 3 (209) | (SMD = −7.56, 95% CI = −10.79 to –4.32, p < 0.00001), in favour of VRG | Low | Risk of bias: 3/3 at low risk of bias (not downgraded)  Inconsistency: I^2^=96% (downgraded)  Indirectness: N/A  Imprecision: n < 400 (downgraded)  Publication Bias: N/A |
| CLBP: ‘VR-based Nintendo vs. control’ (Brea-Gómez et al., 2021) | Pain: ‘VAS, 0–10’ | 4 (155) | (SMD = −0.07, 95% CI = −0.57 to 0.43, p = 0.78), no significant difference | Very low | Risk of bias: 3/4 at high risk of bias (downgraded)  Inconsistency: I^2^=54% (downgraded)  Indirectness: N/A  Imprecision: n < 400 (downgraded)  Publication Bias: N/A |
| CLBP: ‘VR-based horse riding simulator vs. control’ (Brea-Gómez et al., 2021) | Pain: ‘VAS, 0–10’ | 4 (202) | (SMD = −1.68; 95% CI = −2.95 to –0.41, p = 0.009), in favour of VRG | Very low | Risk of bias: 1/4 at high risk of bias (downgraded)  Inconsistency: I^2^=92% (downgraded)  Indirectness: N/A  Imprecision: n < 400 (downgraded)  Publication Bias: N/A |
| CLBP: ‘VR-based horse riding simulator and VR-based Prokin System vs. control’ at intermediate term (Brea-Gómez et al., 2021) | Pain: ‘VAS, 0–10’ | 4 (240) | (SMD = −6.34, 95% CI = −9.12 to –3.56, p < 0.00001), in favour of VRG | Very low | Risk of bias: 1/4 at high risk of bias (downgraded)  Inconsistency: I^2^=97% (downgraded)  Indirectness: N/A  Imprecision: n < 400 (downgraded)  Publication Bias: N/A |
| CLBP: ‘VR vs. control’ (Brea-Gómez et al., 2021) | Pain: ‘VAS, 0–10’ | 11 (569) | (SMD = −1.92, 95% CI = −2.73 to −1.11, p < 0.00001), in favour of VRG | Low | Risk of bias: 4/11 at high risk of bias (downgraded)  Inconsistency: I^2^=93% (downgraded)  Indirectness: N/A  Imprecision: n > 400 (not downgraded)  Publication Bias: NR (not downgraded) |
| CLBP: ‘VR vs. control intervention’ (Brea-Gómez et al., 2021) | Pain: ‘VAS, 0–10’ eight weeks intervention duration. | 6 (258) | (SMD = −0.65, 95% CI = −1.29 to −0.00, p = 0.05), in favour of VRG | Low | Risk of bias: 4/6 at high risk of bias (downgraded)  Inconsistency: I^2^=81% (downgraded)  Indirectness: N/A  Imprecision: n < 400 (downgraded)  Publication Bias: N/A |
| CLBP: VR vs. control (Grassini, 2022) | Pain: ‘present pain intensity scale, NR’ | 2 (136) | (MD = −1.07, 95% CI = −3.37 to 1.24, p = 0.36), no significant difference | Low | Risk of bias: 2/2 at low risk of bias (not downgraded)  Inconsistency: I^2^=96% (downgraded)  Indirectness: N/A  Imprecision: n < 400 (downgraded)  Publication Bias: N/A |
| CLBP: ‘VR vs. control’ (Brea-Gómez et al., 2021) | Kinesiophobia: ‘TSK-17, NR’ | 3 (192) | (MD = −8.96, 95% CI = −17.52 to –0.40, p = 0.04), in favour of VRG | Very low | Risk of bias: 1/3 at high risk of bias (downgraded)  Inconsistency: I^2^=99% (downgraded)  Indirectness: N/A  Imprecision: n < 400 (downgraded)  Publication Bias: N/A |
| CLBP: VR vs. control (Grassini, 2022) | Kinesiophobia: ‘TSK, 0–68’ | 3 (136) | (MD = −9.77, 95% CI = −21.43 to 1.88, p = 0.10), no significant difference | Very low | Risk of bias: 2/3 at high risk of bias (downgraded)  Inconsistency: I^2^=98% (downgraded)  Indirectness: N/A  Imprecision: n < 400 (downgraded)  Publication Bias: N/A |
| CLBP: ‘VR-based Prokin System vs. control for a 4-week intervention’ (Brea-Gómez et al., 2021) | Kinesiophobia: ‘TSK-17, NR’ | 2 (152) | (MD = −12.05, 95% CI = −20.13 to −3.98, p = 0.003), in favour of VRG | Low | Risk of bias: 2/2 at low risk of bias (not downgraded)  Inconsistency: I^2^=98% (downgraded)  Indirectness: N/A  Imprecision: n < 400 (downgraded)  Publication Bias: N/A |
| CLBP: ‘VR vs. control’ at intermediate term (Brea-Gómez et al., 2021) | Kinesiophobia: ‘TSK, NR’ | 2 (149) | (MD = −12.04; 95% CI = −20.58 to –3.49, p = 0.006), in favour of VRG | Low | Risk of bias: 2/2 at low risk of bias (not downgraded)  Inconsistency: I^2^=99% (downgraded)  Indirectness: N/A  Imprecision: n < 400 (downgraded)  Publication Bias: N/A |
| CLBP: ‘VR vs. control group’ at short term (Li et al., 2024) | Kinesiophobia ‘TSK, NR’ | 6 (229) | (MD = −5.46, 95% CI = −9.40 to −1.52, p = 0.007), in favour of VRG. | Very low | Risk of bias: 6/6 at some concerns risk of bias (downgraded)  Inconsistency: I^2^=90% (downgraded)  Indirectness: N/A  Imprecision: n < 400 (downgraded)  Publication Bias: N/A |
| CLBP: ‘VR vs. control group’ at intermediate term (Li et al., 2024) | Kinesiophobia ‘TSK, NR’ | 3 (162) | (MD = −5.66, 95% CI = −12.34 to 1.01, p = 0.10), no significant difference. | Very low | Risk of bias: 3/3 at some concerns risk of bias (downgraded)  Inconsistency: I^2^=96% (downgraded)  Indirectness: N/A  Imprecision: n < 400 (downgraded)  Publication Bias: N/A |
| CLBP: ‘VR vs. control group’ at short term (Li et al., 2024) | Disability ‘Oswestry  Disability Index (ODI), NR’ | 8 (377) | (MD = −11.50, 95% CI = −20.00 to −3.01, p = 0.008), in favour of VRG. | Very low | Risk of bias: 7/8 at some concerns risk of bias (downgraded)  Inconsistency: I^2^=95% (downgraded)  Indirectness: N/A  Imprecision: n < 400 (downgraded)  Publication Bias: N/A |
| CLBP: ‘VR vs. control group’ at intermediate term (Li et al., 2024) | Disability ‘ODI, NR’ | 3 (96) | (MD = −1.28, 95% CI = −4.47 to 1.90, p = 0.43), no significant difference. | Low | Risk of bias: 3/3 at some concerns risk of bias (downgraded)  Inconsistency: I^2^=0% (not downgraded)  Indirectness: N/A  Imprecision: n < 400 (downgraded)  Publication Bias: N/A |
| CLBP: ‘VR-based horse riding simulator vs. control for 4-, 8-, and 12-week intervention duration’ (Brea-Gómez et al., 2021) | Disability: ‘ODI, NR’ | 3 (147) | (MD = −10.46, 95% CI = −30.02 to 9.09, p = 0.29), no significant difference | Very low | Risk of bias: 1/3 at high risk of bias (downgraded)  Inconsistency: I^2^=99% (downgraded)  Indirectness: N/A  Imprecision: n < 400 (downgraded)  Publication Bias: N/A |
| CLBP: ‘VR vs. control’ (Brea-Gómez et al., 2021) | Disability: ‘ODI, NR’ | 3 (147) | (MD = −10.46; 95% CI = −30.02 to 9.09, p = 0.29), no significant difference | Very low | Risk of bias: 1/3 at high risk of bias (downgraded)  Inconsistency: I^2^=99% (downgraded)  Indirectness: N/A  Imprecision: n < 400 (downgraded)  Publication Bias: N/A |
| CLBP: VR vs. control (Grassini, 2022) | Disability: ‘ODI, 0–100’ | 2 (66) | (MD = −0.67, 95% CI = −7.81 to −6.46, p = 0.85), no significant difference | Very low | Risk of bias: 1/2 at high risk of bias (downgraded)  Inconsistency: I^2^=73% (downgraded)  Indirectness: N/A  Imprecision: n < 400 (downgraded)  Publication Bias: N/A. |
| Chronic spinal pain (CSP): VR vs. other interventions (Grassini, 2022) | Pain: ‘VAS, 0–100’ | 3 (112) | (MD = −0.70, 95% CI = −7.88 to −6.47, p = 0.85), no significant difference | Low | Risk of bias: 2/3 at high risk of bias (downgraded)  Inconsistency: I^2^=50% (not downgraded)  Indirectness: N/A  Imprecision: n < 400 (downgraded)  Publication Bias: N/A |
| CSP: ‘VR vs. control’ (Grassini, 2022) | Pain: ‘VAS, 0–100’ | 7 (147) | (MD = −9.10, 95% CI = −17.64 to −0.57, p = 0.04), decreased VAS score in favour of VRG | Very low | Risk of bias: 5/7 at high risk of bias (downgraded)  Inconsistency: I^2^=92% (downgraded)  Indirectness: N/A  Imprecision: n < 400 (downgraded)  Publication Bias: N/A |
| CSP: ‘VR pre-intervention vs post-intervention’ (Grassini, 2022) | Pain: ‘VAS, 0–100’ | 6 (147) | (MD = 29.53, 95% CI = 16.13 to 42.93, p < 0.0001), decreased VAS score in favour of post-intervention | Very low | Risk of bias: 5/6 at high risk of bias (downgraded)  Inconsistency: I^2^=96% (downgraded)  Indirectness: N/A  Imprecision: n < 400 (downgraded)  Publication Bias: N/A |
| CSP: ‘VR vs. control’ (Grassini, 2022) | Kinesiophobia: ‘TSK, 0–68’ | 5 (130) | (MD = −6.00, 95% CI = −14.57 to 2.57, p = 0.17), no significant difference | Very low | Risk of bias: 4/5 at high risk of bias (downgraded)  Inconsistency: I^2^=97% (downgraded)  Indirectness: N/A  Imprecision: n < 400 (downgraded)  Publication Bias: N/A |
| CSP: ‘VR vs. control group’ (Zhang et al., 2024) | Pain: ‘VAS, 0–10’ | 15 (776) | (WMD = −1.63, 95% CI = −2.11 to −1.20, p ˂ 0.001), in favour of VRG. | Low | Risk of bias: 10/15 at high risk of bias (downgraded)  Inconsistency: I^2^=90% (downgraded)  Indirectness: N/A  Imprecision: n > 400 (not downgraded)  Publication Bias: NR (not downgraded) |
| CSP: ‘Immersive VR vs. control group’ (Zhang et al., 2024) | Pain: ‘VAS, 0–10’ | 7 (406) | (WMD = −1.50, 95% CI = −2.45 to −0.55, p ˂ 0.001), in favour of VRG. | Low | Risk of bias: 5/7 at high risk of bias (downgraded)  Inconsistency: I^2^=80% (downgraded)  Indirectness: N/A  Imprecision: n ˃ 400 (not downgraded)  Publication Bias: N/A |
| CSP: ‘Non-immersive VR vs. control group’ (Zhang et al., 2024) | Pain: ‘VAS, 0–10’ | 8 (370) | (WMD = −1.79, 95% CI = −2.31 to −1.27, p ˂ 0.001), in favour of VRG. | Very low | Risk of bias: 5/8 at high risk of bias (downgraded)  Inconsistency: I^2^=91% (downgraded)  Indirectness: N/A  Imprecision: n ˂ 400 (downgraded)  Publication Bias: N/A |
| CSP: ‘VR vs. control group’ (Zhang et al., 2024) | Pain: ‘VAS, 0–10’ (˂ 4 weeks treatment duration) | 2 (119) | (WMD = −1.41, 95% CI = −2.12 to −0.69, p ˂ 0.001), in favour of VRG. | Low | Risk of bias: 2/2 at high risk of bias (downgraded)  Inconsistency: I^2^=0% (not downgraded)  Indirectness: N/A  Imprecision: n ˂ 400 (downgraded)  Publication Bias: N/A |
| CSP: ‘VR vs. control group’ (Zhang et al., 2024) | Pain: ‘VAS, 0–10’ (≥ 4 weeks treatment duration) | 13 (657) | (WMD = −1.65, 95% CI = −2.16 to −1.14, p ˂ 0.001), in favour of VRG. | Low | Risk of bias: 8/13 at high risk of bias (downgraded)  Inconsistency: I^2^=91% (downgraded)  Indirectness: N/A  Imprecision: n ˃ 400 (not downgraded)  Publication Bias: NR |
| CSP: ‘VR vs. control group’ (Zhang et al., 2024) | Kinesiophobia ‘TSK-11, NR’ | 2 (66) | (WMD = −0.81, 95% CI = −4.48 to 2.86, p = 0.66), no significant difference. | Low | Risk of bias: 2/2 at high risk of bias (downgraded)  Inconsistency: I^2^=0 % (downgraded)  Indirectness: N/A  Imprecision: n < 400 (downgraded)  Publication Bias: N/A |
| CSP: ‘VR vs. control group’ (Zhang et al., 2024) | Kinesiophobia ‘TSK-17, NR’ | 2 (96) | (WMD = −9.66, 95% CI = −22.01 to 2.68, p = 0.13), no significant difference. | Very low | Risk of bias: 1/2 at high risk of bias (downgraded)  Inconsistency: I^2^=97% (downgraded)  Indirectness: N/A  Imprecision: n < 400 (downgraded)  Publication Bias: N/A |
| CSP: ‘VR vs. control group’ (Zhang et al., 2024) | Disability ‘NDI, NR’ | 3 (139) | (WMD = −2.66, 95% CI = −5.47 to −0.15, p = 0.06), no significant difference. | Low | Risk of bias: 3/3 at high risk of bias (downgraded)  Inconsistency: I^2^=48% (not downgraded)  Indirectness: N/A  Imprecision: n < 400 (downgraded)  Publication Bias: N/A |

Brea-Gómez, B., Torres-Sánchez, I., Ortiz-Rubio, A., Calvache-Mateo, A., Cabrera-Martos, I., López-López, L., & Valenza, M. C. (2021). Virtual reality in the treatment of adults with chronic low back pain: a systematic review and meta-analysis of randomized clinical trials. *Int J Environ Res Public Health, 18*(22), 11806. doi:<https://doi.org/10.3390/ijerph182211806>

Grassini, S. (2022). Virtual reality assisted non-pharmacological treatments in chronic pain management: a systematic review and quantitative meta-analysis. *Int J Environ Res Public Health, 19*(7), 4071. doi:<https://doi.org/10.3390/ijerph19074071>

Hao, J., He, Z., Chen, Z., & Remis, A. (2024). Virtual reality training versus conventional rehabilitation for chronic neck pain: a systematic review and meta-analysis. *PM&R*, 1-11. doi:<https://doi.org/10.1002/pmrj.13158>

Kumar, V., Vatkar, A. J., Kataria, M., Dhatt, S. S., & Baburaj, V. (2024). Virtual reality is effective in the management of chronic low back ache in adults: a systematic review and meta-analysis of randomized controlled trials. *Eur Spine J, 33*(2), 474-480. doi:<https://doi.org/10.1007/s00586-023-08040-5>

Li, R., Li, Y., Kong, Y., Li, H., Hu, D., Fu, C., & Wei, Q. (2024). Virtual reality-based training in chronic low back pain: systematic review and meta-analysis of randomized controlled trials. *J Med Internet Res, 26*(1), e45406. doi:<https://doi.org/10.2196/45406>

Ye, G., Koh, R. G. L., Jaiswal, K., Soomal, H., & Kumbhare, D. (2023). The use of virtual reality in the rehabilitation of chronic nonspecific neck pain: a systematic review and meta-analysis. *Clin J Pain, 39*(9), 491-500. doi:<https://doi.org/10.1097/AJP.0000000000001134>

Zhang, T. T., Li, X., Zhou, X., Zhan, L. X., Wu, F., Huang, Z. F., . . . Du, Q. (2024). Virtual reality therapy for the management of chronic spinal pain: systematicu review and meta-analysis. *JMIR Serious Games 12*. doi:<https://doi.org/10.2196/50089>
