# Supplementary material for: Virtual Reality for Patients With Chronic Musculoskeletal Pain and Disability: An Umbrella Review of Systematic Reviews
Source: Health Sci Rep. 2025 Aug 12;8(8):e71163. doi: 10.1002/hsr2.71163 (PMC12343317; doi:10.1002/hsr2.71163)
Supplement: Supplementary file 6 — S6 File. Virtual Reality and Control Group Interventions. [file HSR2-8-e71163-s004.docx]

| Conditions | Primary studies | Virtual reality (VR) intervention | List of control intervention | VR intervention number of sessions/durations of intervention | Country | Included reviews |
| --- | --- | --- | --- | --- | --- | --- |
| Chronic neck pain | Rezaei, Razeghi, Ebrahimi, Kayedi, and Rezaeian Zadeh (2019) | VR-based computer game (non-immersive) | Conventional proprioception training | Eight 21-min sessions over 4 weeks | Iran | Grassini (2022); Hao, He, Chen, and Remis (2024); Ye, Koh, Jaiswal, Soomal, and Kumbhare (2023) |
|  | Bahat et al. (2018) | VR-based Oculus Rift head-mounted display (HMD) (immersive) | Kinematic training using a head-mounted laser | Four 5-min sessions/day, 4 times/week for 4 weeks | Australia | Grassini (2022); Hao et al. (2024); Zhang et al. (2024) |
|  | Tejera et al. (2020) | VR-based Vox Play glasses (non-immersive) | Care as usual | Two (unclear time) sessions/week for 4 weeks | Spain | Grassini (2022); Hao et al. (2024); Ye et al. (2023); Zhang et al. (2024) |
|  | Bahat, Takasaki, Chen, Bet-Or, and Treleaven (2015) | VR-based Oculus Rift HMD (immersive) + kinematic training using a head-mounted laser | Kinematic training using a head-mounted laser | Four to six 30-min sessions over 5 weeks | Australia | Hao et al. (2024) |
|  | Cetin, Kose, and Oge (2022) | VR-based Oculus Rift HMD (immersive) + motor control exercises | Motor control exercises | Three 20-min sessions over 6 weeks | Turkey | Hao et al. (2024); Ye et al. (2023); Zhang et al. (2024) |
|  | Nusser, Knapp, Kramer, and Krischak (2021) | VR-based 5DT HMD 800 (immersive) + specific sensorimotor training | General sensorimotor training + care as usual | Six 20-min sessions over 3 weeks | Germany | Hao et al. (2024); Ye et al. (2023); Zhang et al. (2024) |
|  | Glavare, Stålnacke, Häger, and Löfgren (2021) | VR-based mobile phone in VR glasses (immersive) + physical activity | N/A | Two 10-30-min sessions/week for 6 weeks. | Sweden | Ye et al. (2023) |
| Chronic low back pain | Kim, Min, Kim, and Lee (2014) | VR-based Nintendo Wii Fit yoga program (non-immersive) | Trunk-stabilising exercises + physiotherapy | Three 30-min sessions/week for 4 weeks | South Korea | Li et al. (2024) |
|  | Park, Lee, and Ko (2013) | VR-based Nintendo Wii exercises (non-immersive) + physiotherapy | Physiotherapy | Three 30-min sessions/week for 8 weeks | South Korea | Brea-Gómez et al. (2021); Li et al. (2024); Zhang et al. (2024) |
|  | Oh et al. (2014) | VR-based horse-riding simulator (non-immersive) | No intervention | Five 15-, 20-, and 35-min sessions/week for 8 weeks | South Korea | Brea-Gómez et al. (2021); Li et al. (2024) |
|  | Yoo et al. (2014) | VR-based horse-riding simulator (non-immersive) | No intervention | Three 10-, 20-, 30-, and 40-min sessions/week for 8 weeks | South Korea | Brea-Gómez et al. (2021); Li et al. (2024) |
|  | Monteiro-Junior et al. (2015) | VR-based Wii Fit Plus workout (non-immersive) + core exercises and strength training | Core exercises and strength training | Three 30-min sessions/week for 8 weeks | Brazil | Brea-Gómez et al. (2021); Li et al. (2024); Zhang et al. (2024) |
|  | Chen, Kim, Kim, Lee, and HwangBo (2016) | VR-based horse-riding simulator (non-immersive) + lumbar strengthening exercise | Lumbar strengthening exercise | Three 15-min sessions/week for 4 weeks | South Korea | Brea-Gómez et al. (2021) |
|  | Zadro et al. (2019) | VR-based Wii Fit U exercises (non-immersive) | No intervention | Three 60-min sessions/week for 8 weeks | Australia | Brea-Gómez et al. (2021); Li et al. (2024) |
|  | Kim, Lee, Oh, Kim, and Yoon (2020) | VR-based horse-riding simulator (non-immersive) | Stabilisation exercise | Two 30-min sessions/week for 8 weeks | South Korea | Brea-Gómez et al. (2021); Li et al. (2024) |
|  | Nambi et al. (2020) | VR-based Prokin System (non-immersive) + physiotherapy | Conventional balance training + physiotherapy | Five 30-min sessions/week for 4 weeks | Saudi Arabia | Brea-Gómez et al. (2021); Kumar, Vatkar, Kataria, Dhatt, and Baburaj (2024); Li et al. (2024); Zhang et al. (2024) |
|  | Nambi, Abdelbasset, and Alqahatani (2021) | VR-based Prokin System (non-immersive) + physiotherapy | Conventional balance training + physiotherapy | Five 30-min sessions/week for 4 weeks | Saudi Arabia | Zhang et al. (2024) |
|  | Nambi, Abdelbasset, Alrawaili, et al. (2021) | VR-based Prokin System (non-immersive) + physiotherapy | Conventional balance training + physiotherapy | Five 30-min sessions/week for 4 weeks | Saudi Arabia | Brea-Gómez et al. (2021); Grassini (2022); Li et al. (2024) |
|  | Nambi, Abdelbasset, Alsubaie, et al. (2021) | VR-based Prokin System (non-immersive) + physiotherapy | Conventional balance training + physiotherapy | Five 30-min sessions/week for 4 weeks | Saudi Arabia | Brea-Gómez et al. (2021); Li et al. (2024); Zhang et al. (2024) |
|  | Nambi, Abdelbasset, Elsayed, et al. (2021) | VR-based Prokin System (non-immersive) + physiotherapy | Conventional balance training + physiotherapy | Five 30-min sessions/week for 4 weeks | Saudi Arabia | Li et al. (2024); Zhang et al. (2024) |
|  | Nambi et al. (2022) | VR-based Prokin System (non-immersive) + physiotherapy | Conventional balance training + physiotherapy | Five 30-min sessions/week for 4 weeks | Saudi Arabia | Zhang et al. (2024) |
|  | Park, Park, Min, Kim, and Jee (2020) | VR-based horse-riding simulator (non-immersive) | Watching video | Three 15-min sessions/week for 12 weeks | South Korea | Brea-Gómez et al. (2021); Li et al. (2024) |
|  | Tomruk, Bilge, and Erbayraktar (2020) | VR-based biofeedback program via glasses with the Biodex Balance System (non-immersive) | Traditional training | Two 30-min sessions/week for 12 weeks | Turkey | Brea-Gómez et al. (2021) |
|  | Garcia et al. (2021) | VR-based Pico (EaseVRx) HMD (immersive) | Sham VR | Seven 2–16-min sessions/week for 8 weeks | United States | Brea-Gómez et al. (2021); Kumar et al. (2024); Li et al. (2024); Zhang et al. (2024) |
|  | Sato et al. (2021) | VR-based Nintendo Ring Fit Adventure (non-immersive) | Oral treatment | One 40-min session/week for 8 weeks | Japan | Brea-Gómez et al. (2021) |
|  | Thomas, France, Applegate, Leitkam, and Walkowski (2016) | VR-based Samsung 3D eyeglasses (non-immersive) | Care as usual | Three 15-min sessions over 3 days | United States | Grassini (2022) |
|  | Li et al. (2021) | VR-based Kinetic Xbox (non-immersive) + magnetic therapy | Conventional thermal magnetic therapy | Six 3-min sessions/day, 5 times/week for 2 weeks | China | Grassini (2022); Li et al. (2024) |
|  | Yilmaz Yelvar et al. (2017) | VR-based eyeglasses video of virtual walking (non-immersive) + care as usual | Care as usual | Five 35-min sessions/week for 2 weeks | Turkey | Grassini (2022); Li et al. (2024) |
|  | Matheve, Bogaerts, and Timmermans (2020) | VR-based Valedo^®^Pro (non-immersive) + exercises | Care as usual | One 4-min session | Belgium | Grassini (2022); Kumar et al. (2024); Zhang et al. (2024) |
|  | Darnall, Krishnamurthy, Tsuei, and Minor (2020) | VR-based Oculus GO HMD (immersive) | Audio Instruction Programme | One 1–15-min session/day for 21 days | United States | Grassini (2022); Kumar et al. (2024) |
|  | Afzal et al. (2022) | VR-based Kinetic Xbox (non-immersive) + physiotherapy | Back strengthening exercise + physiotherapy | Three 10-min sessions/week for 4 weeks | Pakistan | Kumar et al. (2024); Li et al. (2024); Zhang et al. (2024) |
|  | Yalfani, Abedi, and Raeisi (2022) | VR-based HTC-Vive (immersive) | Daily life routines | Three 30-min sessions/week for 8 weeks | Iran | Kumar et al. (2024); Li et al. (2024); Zhang et al. (2024) |
|  | Stamm, Dahms, Reithinger, Ruß, and Müller-Werdan (2022) | VR-based HTC-Vive (immersive) + psychoeducation | Chair-based exercises + psychoeducation | Three 30-min sessions/week for 4 weeks | Germany | Zhang et al. (2024) |
|  | Eccleston et al. (2022) | VR-based Oculus Quest HMD (immersive) | Care as usual | Five 15-60-min sessions/week for 6-8 weeks | Finland | Kumar et al. (2024); Li et al. (2024) |
|  | Groenveld et al. (2023) | VR-based Oculus GO HMD (immersive) | Daily life routines | Three 10-30-min sessions/day, 7 times/week for 4 weeks | finland | Li et al. (2024) |
|  | Meinke, Peters, Knols, Swanenburg, and Karlen (2022) | VR-based Valedo®Pro (non-immersive) | Daily life routines | Three 20-min sessions/week for 3 weeks | Switzerland | Li et al. (2024) |

Afzal, M. W., Ahmad, A., Bandpei, M. M., Gilani, S. A., Hanif, A., & Waqas, M. S. (2022). Effects of virtual reality exercises and routine physical therapy on pain intensity and functional disability in patients with chronic low back pain. *J Pak Med Assoc, 72*(3), 413-417. doi:<https://doi.org/10.47391/jpma.3424>

Bahat, H. S., Croft, K., Carter, C., Hoddinott, A., Sprecher, E., & Treleaven, J. (2018). Remote kinematic training for patients with chronic neck pain: a randomised controlled trial. *Eur Spine J, 27*(6), 1309-1323. doi:<https://doi.org/10.1007/s00586-017-5323-0>

Bahat, H. S., Takasaki, H., Chen, X., Bet-Or, Y., & Treleaven, J. (2015). Cervical kinematic training with and without interactive VR training for chronic neck pain–a randomized clinical trial. *Man Ther, 20*(1), 68-78. doi:<https://doi.org/10.1016/j.math.2014.06.008>

Brea-Gómez, B., Torres-Sánchez, I., Ortiz-Rubio, A., Calvache-Mateo, A., Cabrera-Martos, I., López-López, L., & Valenza, M. C. (2021). Virtual reality in the treatment of adults with chronic low back pain: a systematic review and meta-analysis of randomized clinical trials. *Int J Environ Res Public Health, 18*(22), 11806. doi:<https://doi.org/10.3390/ijerph182211806>

Cetin, H., Kose, N., & Oge, H. K. (2022). Virtual reality and motor control exercises to treat chronic neck pain: a randomized controlled trial. *Musculoskelet Sci Pract, 62*, 102636. doi:<https://doi.org/10.1016/j.msksp.2022.102636>

Chen, S.-Y., Kim, S.-K., Kim, K.-H., Lee, I.-S., & HwangBo, G. (2016). Effects of horse riding simulator on pain, oswestry disability index and balance in adults with nonspecific chronic low back pain. *J Korean Soc Phys Med, 11*(4), 79-84. doi:<https://doi.org/10.13066/kspm.2016.11.4.79>

Darnall, B. D., Krishnamurthy, P., Tsuei, J., & Minor, J. D. (2020). Self-administered skills-based virtual reality intervention for chronic pain: randomized controlled pilot study. *JMIR Form Res, 4*(7), e17293. doi:<https://doi.org/10.2196/17293>

Eccleston, C., Fisher, E., Liikkanen, S., Sarapohja, T., Stenfors, C., Jääskeläinen, S. K., . . . Bratty, J. R. (2022). A prospective, double-blind, pilot, randomized, controlled trial of an “embodied” virtual reality intervention for adults with low back pain. *Pain, 163*(9), 1700-1715. doi:<https://doi.org/10.1097/j.pain.0000000000002617>

Garcia, L., Birckhead, B., Krishnamurthy, P., Sackman, J., Mackey, I., Louis, R., . . . Darnall, B. (2021). An 8-week self-administered at-home behavioral skills-based virtual reality program for chronic low back pain: double-blind, randomized, placebo-controlled trial conducted during COVID-19. *J Med Internet Res, 23*(2), e26292. doi:<https://doi.org/10.2196/26292>

Glavare, M., Stålnacke, B. M., Häger, C. K., & Löfgren, M. (2021). Virtual reality exercises in an interdisciplinary rehabilitation programme for persons with chronic neck pain: a feasibility study. *J Rehabil Med Clin Commun, 4*, 1000067. doi:<https://doi.org/10.2340/20030711-1000067>

Grassini, S. (2022). Virtual reality assisted non-pharmacological treatments in chronic pain management: a systematic review and quantitative meta-analysis. *Int J Environ Res Public Health, 19*(7), 4071. doi:<https://doi.org/10.3390/ijerph19074071>

Groenveld, T. D., Smits, M. L. M., Knoop, J., Kallewaard, J. W., Staal, J. B., de Vries, M., & van Goor, H. (2023). Effect of a behavioral therapy-based

virtual reality application on quality of life in chronic low back pain. *Clin J Pain, 39*(6). doi:<https://doi.org/10.1097/AJP.0000000000001110>

Hao, J., He, Z., Chen, Z., & Remis, A. (2024). Virtual reality training versus conventional rehabilitation for chronic neck pain: a systematic review and meta-analysis. *PM&R*, 1-11. doi:<https://doi.org/10.1002/pmrj.13158>

Kim, S., Min, W., Kim, J., & Lee, B. (2014). The effects of VR-based Wii Fit yoga on physical function in middle-aged female LBP patients. *J Phys Ther Sci, 26*(4), 549-552. doi:<https://doi.org/10.1589/jpts.26.549>

Kim, T., Lee, J., Oh, S., Kim, S., & Yoon, B. (2020). Effectiveness of simulated horseback riding for patients with chronic low back pain: a randomized controlled trial. *J Sport Rehabil, 29*(2), 179-185. doi:<https://doi.org/10.1123/jsr.2018-0252>

Kumar, V., Vatkar, A. J., Kataria, M., Dhatt, S. S., & Baburaj, V. (2024). Virtual reality is effective in the management of chronic low back ache in adults: a systematic review and meta-analysis of randomized controlled trials. *Eur Spine J, 33*(2), 474-480. doi:<https://doi.org/10.1007/s00586-023-08040-5>

Li, R., Li, Y., Kong, Y., Li, H., Hu, D., Fu, C., & Wei, Q. (2024). Virtual reality-based training in chronic low back pain: systematic review and meta-analysis of randomized controlled trials. *J Med Internet Res, 26*(1), e45406. doi:<https://doi.org/10.2196/45406>

Li, Z., Yu, Q., Luo, H., Liang, W., Li, X., Ge, L., . . . Wang, C. (2021). The effect of virtual reality training on anticipatory postural adjustments in patients with chronic nonspecific low back pain: a preliminary study. *Neural Plast, 2021*. doi:<https://doi.org/10.1155/2021/9975862>

Matheve, T., Bogaerts, K., & Timmermans, A. (2020). Virtual reality distraction induces hypoalgesia in patients with chronic low back pain: a randomized controlled trial. *J Neuroeng Rehabil, 17*(1), 55. doi:<https://doi.org/10.1186/s12984-020-00688-0>

Meinke, A., Peters, R., Knols, R. H., Swanenburg, J., & Karlen, W. (2022). Feedback on trunk movements from an electronic game to improve postural balance in people with nonspecific low back pain: pilot randomized controlled trial. *JMIR Serious Games, 10*(2), e31685. doi:<https://doi.org/10.2196/31685>

Monteiro-Junior, R. S., de Souza, C., Lattari, E., Rocha, N., Mura, G., Machado, S., & da Silva, E. B. (2015). Wii-workouts on chronic pain, physical capabilities and mood of older women: a randomized controlled double blind trial. *CNS Neurol Disord Drug Targets, 14*(9), 1157-1164.

Nambi, G., Abdelbasset, K., Elsayed, S., Alrawaili, S., Abodonya, A., Saleh, A., & Elnegamy, T. (2020). Comparative effects of isokinetic training and virtual reality training on sports performances in university football players with chronic low back pain-randomized controlled study. *Evid Based Complement Alternat Med, 2020*, 2981273. doi:<https://doi.org/10.1155/2020/2981273>

Nambi, G., Abdelbasset, W., & Alqahatani, B. (2021). Radiological (magnetic resonance image and ultrasound) and biochemical effects of virtual reality training on balance training in football players with chronic low back pain: a randomized controlled study. *J Back Musculoskelet Rehabil, 34*, 269-277. doi:<https://doi.org/10.3233/BMR-191657>

Nambi, G., Abdelbasset, W., Alrawaili, S., Alsubaie, S., Abodonya, A., & Saleh, A. (2021). Virtual reality or isokinetic training; its effect on pain, kinesiophobia and serum stress hormones in chronic low back pain: a randomized controlled trial. *Technol Health Care, 29*, 155-166. doi:<https://doi.org/10.3233/THC-202301>

Nambi, G., Abdelbasset, W., Alsubaie, S., Saleh, A., Verma, A., Abdelaziz, M., & Alkathiry, A. (2021). Short-term psychological and hormonal effects of virtual reality training on chronic low back pain in soccer players. *J Sport Rehabil, 30*(6), 884-893. doi:<https://doi.org/10.1123/jsr.2020-0075>

Nambi, G., Abdelbasset, W., Elsayed, S., Verma, A., George, J., & Saleh, A. (2021). Clinical and physical efficiency of virtual reality games in soccer players with low back pain. *Rev Bras Med Esporte, 27*(6), 597-602. doi:<https://doi.org/10.1590/1517-8692202127062021_0034>

Nambi, G., Alghadier, M., Kashoo, F., Aldhafian, O., Nwihadh, N., Saleh, A., . . . Ismail, M. (2022). Effects of virtual reality exercises versus isokinetic exercises in comparison with conventional exercises on the imaging findings and inflammatory biomarker changes in soccer players with non specific low back pain: a randomized controlled trial. *Int J Environ Res Public Health, 20*(1), 524. doi:<https://doi.org/10.3390/ijerph20010524>

Nusser, M., Knapp, S., Kramer, M., & Krischak, G. (2021). Effects of virtual reality-based neck-specific sensorimotor training in patients with chronic neck pain: a randomized controlled pilot trial. *J Rehabil Med, 53*(2), jrm00151. doi:<https://doi.org/10.2340/16501977-2786>

Oh, H.-W., Lee, M.-G., Jang, J.-Y., Jin, J.-J., Cha, J.-Y., Jin, Y.-Y., & Jee, Y.-S. (2014). Time-effects of horse simulator exercise on psychophysiological responses in men with chronic low back pain. *Isokinet Exerc Sci, 22*(2), 153-163. doi:<https://doi.org/10.3233/IES-140533>

Park, J. H., Lee, S. H., & Ko, D. S. (2013). The effects of the Nintendo Wii exercise program on chronic work-related low back pain in industrial workers. *J Phys Ther Sci, 25*(8), 985-988. doi:<https://doi.org/10.1589/jpts.25.985>

Park, S., Park, S., Min, S., Kim, C.-J., & Jee, Y.-S. (2020). A randomized controlled trial investigating the effects of equine simulator riding on low back pain, morphological changes, and trunk musculature in elderly women. *Medicina, 56*(11), 610. doi:<https://doi.org/10.3390/medicina56110610>

Rezaei, I., Razeghi, M., Ebrahimi, S., Kayedi, S., & Rezaeian Zadeh, A. (2019). A novel virtual reality technique (Cervigame®) compared to conventional proprioceptive training to treat neck pain: a randomized controlled trial. *J Biomed Phys Eng, 9*(3), 355-366. doi:<https://doi.org/10.31661/jbpe.v0i0.556>

Sato, T., Shimizu, K., Shiko, Y., Kawasaki, Y., Orita, S., Inage, K., . . . Enomoto, K. (2021). Effects of Nintendo Ring Fit Adventure exergame on pain and psychological factors in patients with chronic low back pain. *Games Health J, 10*(3), 158-164. doi:<https://doi.org/10.1089/g4h.2020.0180>

Stamm, O., Dahms, R., Reithinger, N., Ruß, A., & Müller-Werdan, U. (2022). Virtual reality exergame for supplementing multimodal pain therapy in older adults with chronic back pain: a randomized controlled pilot study. *Virtual Real, 26*(4), 1291-1305. doi:<https://doi.org/10.1007/s10055-022-00629-3>

Tejera, D. M., Beltran-Alacreu, H., Cano-de-la-Cuerda, R., Leon Hernández, J. V., Martín-Pintado-Zugasti, A., Calvo-Lobo, C., . . . Fernández-Carnero, J. (2020). Effects of virtual reality versus exercise on pain, functional, somatosensory and psychosocial outcomes in patients with non-specific chronic neck pain: a randomized clinical trial. *Int J Environ Res Public Health, 17*(16), 5950. doi:<https://doi.org/10.3390/ijerph17165950>

Thomas, J., France, C., Applegate, M., Leitkam, S., & Walkowski, S. (2016). Feasibility and safety of a virtual reality dodgeball intervention for chronic low back pain: a randomized clinical trial. *Pain, 17*(12), 1302-1317. doi:<https://doi.org/10.1016/j.jpain.2016.08.011>

Tomruk, M. S., Bilge, K., & Erbayraktar, R. S. (2020). The effect of computer-based training on postural control in patients with chronic low back pain: a randomized controlled trial. *J Basic Clin Health Sci, 4*(3), 329-334. doi:<https://doi.org/10.30621/jbachs.2020.1150>

Yalfani, A., Abedi, M., & Raeisi, Z. (2022). Effects of an 8-week virtual reality training program on pain, fall risk, and quality of life in elderly women with chronic low back pain: double-blind randomized clinical trial. *Games Health J, 11*(2), 85-92. doi:<https://doi.org/10.1089/g4h.2021.0175>

Ye, G., Koh, R. G. L., Jaiswal, K., Soomal, H., & Kumbhare, D. (2023). The use of virtual reality in the rehabilitation of chronic nonspecific neck pain: a systematic review and meta-analysis. *Clin J Pain, 39*(9), 491-500. doi:<https://doi.org/10.1097/AJP.0000000000001134>

Yilmaz Yelvar, G. D., Çırak, Y., Dalkılınç, M., Parlak Demir, Y., Guner, Z., & Boydak, A. (2017). Is physiotherapy integrated virtual walking effective on pain, function, and kinesiophobia in patients with non-specific low-back pain? Randomised controlled trial. *Eur Spine J, 26*(2), 538-545. doi:<https://doi.org/10.1007/s00586-016-4892-7>

Yoo, J. H., Kim, S. E., Lee, M. G., Jin, J. J., Hong, J., Choi, Y. T., . . . Jee, Y. S. (2014). The effect of horse simulator riding on visual analogue scale, body composition and trunk strength in the patients with chronic low back pain. *Int J Clin Pract, 68*(8), 941-949. doi:<https://doi.org/10.1111/ijcp.12414>

Zadro, J. R., Shirley, D., Simic, M., Mousavi, S. J., Ceprnja, D., Maka, K., . . . Ferreira, P. (2019). Video-game–based exercises for older people with chronic low back pain: a randomized controlledtable trial (GAMEBACK). *Phys Ther, 99*(1), 14-27. doi:<https://doi.org/10.1093/ptj/pzy112>

Zhang, T. T., Li, X., Zhou, X., Zhan, L. X., Wu, F., Huang, Z. F., . . . Du, Q. (2024). Virtual reality therapy for the management of chronic spinal pain: systematic review and meta-analysis. *JMIR Serious Games 12*. doi:<https://doi.org/10.2196/50089>
